# Endothelial, pericyte and tumor cell expression in glioblastoma identifies fibroblast activation protein (FAP) as an excellent target for immunotherapy

**DOI:** 10.1002/cti2.1191

**Published:** 2020-10-14

**Authors:** Lisa M Ebert, Wenbo Yu, Tessa Gargett, John Toubia, Paris M Kollis, Melinda N Tea, Brenton W Ebert, Cedric Bardy, Mark van den Hurk, Claudine S Bonder, Jim Manavis, Kathleen S Ensbey, Mariana Oksdath Mansilla, Kaitlin G Scheer, Sally L Perrin, Rebecca J Ormsby, Santosh Poonnoose, Barbara Koszyca, Stuart M Pitson, Bryan W Day, Guillermo A Gomez, Michael P Brown

**Affiliations:** ^1^ Centre for Cancer Biology SA Pathology and University of South Australia Adelaide Australia; ^2^ Adelaide Medical School University of Adelaide Adelaide Australia; ^3^ South Australian Health and Medical Research Institute (SAHMRI) Adelaide Australia; ^4^ College of Medicine & Public Health Flinders University Adelaide Australia; ^5^ Department of Cell and Molecular Biology Sid Faithfull Brain Cancer Laboratory QIMR Berghofer Medical Research Institute Brisbane QLD Australia; ^6^ Clinical and Health Sciences University of South Australia Adelaide Australia; ^7^ Department of Neurosurgery Flinders Medical Centre Bedford Park Australia; ^8^ Department of Anatomical Pathology SA Pathology Adelaide Australia; ^9^ Faculty of Health Queensland University of Technology Brisbane QLD Australia; ^10^ Faculty of Medicine The University of Queensland Brisbane QLD Australia; ^11^ Cancer Clinical Trials Unit Royal Adelaide Hospital Adelaide Australia

**Keywords:** blood vessels, fibroblast activation protein, glioblastoma, immunotherapy, scRNAseq, target antigen

## Abstract

**Objectives:**

Targeted immunotherapies such as chimeric antigen receptor (CAR)‐T cells are emerging as attractive treatment options for glioblastoma, but rely on identification of a suitable tumor antigen. We validated a new target antigen for glioblastoma, fibroblast activation protein (FAP), by undertaking a detailed expression study of human samples.

**Methods:**

Glioblastoma and normal tissues were assessed using immunostaining, supported by analyses of published transcriptomic datasets. Short‐term cultures of glioma neural stem (GNS) cells were compared to cultures of healthy astrocytes and neurons using flow cytometry. Glioblastoma tissues were dissociated and analysed by high‐parameter flow cytometry and single‐cell transcriptomics (scRNAseq).

**Results:**

Compared to normal brain, FAP was overexpressed at the gene and protein level in a large percentage of glioblastoma tissues, with highest levels of expression associated with poorer prognosis. *FAP* was also overexpressed in several paediatric brain cancers. FAP was commonly expressed by cultured GNS cells but absent from normal neurons and astrocytes. Within glioblastoma tissues, the strongest expression of FAP was around blood vessels. In fact, almost every tumor vessel was highlighted by FAP expression, whereas normal tissue vessels and cultured endothelial cells (ECs) lacked expression. Single‐cell analyses of dissociated tumors facilitated a detailed characterisation of the main cellular components of the glioblastoma microenvironment and revealed that vessel‐localised FAP is because of expression on both ECs and pericytes.

**Conclusion:**

Fibroblast activation protein is expressed by multiple cell types within glioblastoma, highlighting it as an ideal immunotherapy antigen to target destruction of both tumor cells and their supporting vascular network.

## Graphical abstract

This detailed analysis of the expression patterns of fibroblast activation protein (FAP) reveals it to be an important new target antigen for immunotherapy of glioblastoma. Immunostaining of tissue sections, flow cytometry and single‐cell RNA sequencing reveal broad expression of FAP by not only glioblastoma tumour cells but also endothelial cells and pericytes within the tumour microenvironment.

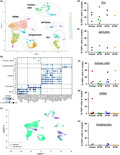

## Introduction

Glioblastoma is the most common and lethal type of primary brain tumor.^1^ Standard first‐line treatment, using debulking surgery followed by radiotherapy and temozolomide chemotherapy, offers limited efficacy and extends median survival by only a matter of months. There is no standard second‐line treatment and none that extends overall survival. Median survival time from diagnosis is only 14.6 months, and the 5‐year survival rate is ~10%. Hence, the unmet clinical need in this disease is great and the development of new therapeutic approaches remains an area of active investigation.

In this regard, targeted immunotherapy approaches may hold particular promise. This includes chimeric antigen receptor (CAR)‐T cell therapies, bispecific T‐cell engagers (BiTEs) and payload‐conjugated monoclonal antibodies, such as antibody–drug conjugates (ADC). All of these approaches take advantage of surface antigens expressed on tumor cells to direct engineered components of the immune system specifically towards these cells, resulting in highly targeted tumor cell killing. But a key requirement for the success of such approaches is the identification of an appropriate tumor antigen. Ideally, this antigen will be uniformly expressed by the tumor cells, and possibly by other supporting cell types within the tumor microenvironment, yet it shows minimal or no expression by any healthy cells. And in the absence of a ‘universal’ tumor antigen, an appropriate target antigen needs to be identified for each cancer type.

Several molecules have been proposed as tumor antigens for targeted immunotherapy of glioblastoma. These include HER2, IL13Rα2, EphA2, EphA3 and mutant forms of the EGFR.^1^, ^2^, ^3^, ^4^, ^5^ Although encouraging results have been obtained in both preclinical and early‐phase clinical studies, immunotherapy approaches targeting these antigens suffer from several drawbacks. First, some of the proposed antigens (e.g. HER2 and EphA2) show significant expression on healthy tissues, which can lead to serious on‐target, off‐tumor toxicity.^6^, ^7^ Second, none of these antigens is uniformly expressed by glioblastoma cells. This reduces treatment efficacy by targeting only a fraction of the tumor cells and allows for outgrowth of antigen‐negative tumors even when the therapy is initially effective.^1^, ^8^

There are several ways to improve the efficacy of immunotherapy targeting for glioblastoma. One option is to adopt combinatorial approaches that allow for simultaneous targeting of multiple antigens. For example, CAR‐T cells can be engineered to express multiple individual CARs, or tandem CARs containing more than a single antigen‐binding domain, to broaden their specificity.^2^, ^9^ BiTEs can be modified to contain multiple tumor antigen‐binding domains,^10^ or CAR‐T cells can be engineered to secrete BiTEs with a distinct antigen specificity to the CAR.^4^

A second option is to identify better antigens that allow for efficient eradication of the tumor using noncombinatorial approaches. Indeed, the identification of CD19 as a target antigen for B‐cell leukaemias and lymphomas has allowed the development of FDA‐approved CD19‐targeting CAR‐T cell therapies that have been used to successfully treat thousands of patients, many of whom remain disease‐free several years after treatment, all via targeting a single antigen.^11^ The identification of an antigen with equivalent expression patterns in glioblastoma could be the first key step in developing a similarly successful treatment for these patients. Of note, the growth of solid tumors such as glioblastoma depends on the integrity of stromal elements within the tumor microenvironment,^12^ raising the possibility of targeting an antigen expressed on these essential stromal cell types instead of – or in addition to – the cancer cells themselves. However, immunotherapy approaches based on targeting the glioblastoma microenvironment are yet to be developed, and indeed, the stromal components of glioblastoma tumors remain to be fully characterised.

Fibroblast activation protein (FAP; also known as seprase) is a surface‐expressed proteolytic enzyme that has been identified as a promising immunotherapy target antigen for epithelial carcinomas such as prostate, lung and pancreatic cancer, and mesothelioma.^13^, ^14^, ^15^, ^16^, ^17^ The interest in FAP as an immunotherapy target for these diverse cancers stems from its broad expression on cancer‐associated fibroblasts (CAFs), which are a major component of the stromal microenvironment of carcinomas.^18^, ^19^ In contrast, expression of FAP on healthy cells and tissues is largely reported to be minimal,^17^, ^18^, ^20^, ^21^, ^22^ although there are conflicting observations.^23^, ^24^ The expression of FAP in carcinomas is generally limited to the stromal CAF population, with FAP being largely absent from the tumor cells themselves. However, we noted with interest reports suggesting that, in glioblastoma, FAP can be expressed by the tumor cells themselves, as well as an unidentified stromal population within the tumor microenvironment.^22^, ^25^, ^26^

Here, we have undertaken a detailed analysis of the expression patterns of FAP in tumor and normal tissues to assess its suitability as a target antigen for immunotherapy of glioblastoma. We aimed for the first time to broadly assess the inter‐ and intratumor heterogeneity in FAP expression; to precisely define the cell types within tumors that express FAP; and to compare glioblastoma to healthy tissues. We found that FAP was consistently overexpressed in a large proportion of patient tumors and patient‐derived glioblastoma cultures compared to normal tissue. Moreover, we have used confocal microscopy, flow cytometry and single‐cell transcriptomics to demonstrate that FAP is expressed by multiple cell types in the glioblastoma microenvironment, with expression on ECs and pericytes as well as the tumor cells. Expression on and around blood vessels was particularly striking, and almost ubiquitous. Together, our findings reveal FAP as a promising new target antigen for immunotherapy of glioblastoma, potentially allowing not only destruction of tumor cells but also effective elimination of their supporting vascular network.

## Results

### Transcriptomic analyses reveal that FAP is overexpressed in glioblastoma compared to normal brain, with limited expression in other normal tissues

**Figure 1:**
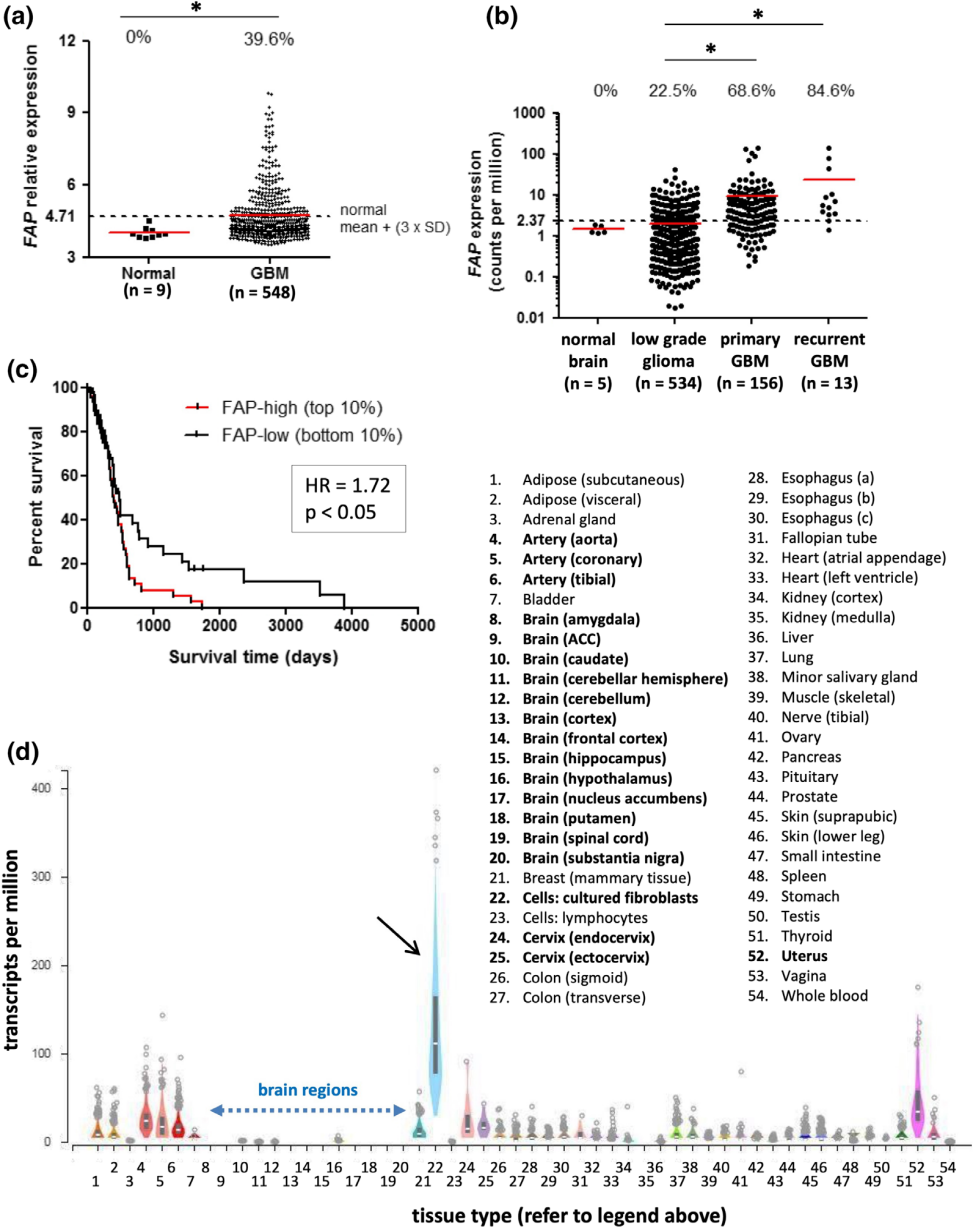
*FAP* expression in transcriptomic analyses of glioblastoma and normal tissues. **(a, b)** *FAP* gene expression values from TCGA microarray (**a**) and RNAseq (**b**) datasets. The expression value for each tissue sample is shown. Red lines represent the median of each group, while dotted lines represent the threshold for *FAP* expression, based on [mean + (3 × SD)] of the respective normal brain dataset. The proportion of samples in each group with expression above the threshold is indicated at the top of the graphs. In **a**, groups were compared by the Mann–Whitney *U*‐test (*P* < 0.01). In **b**, all groups were compared using the Kruskal–Wallis test (*P* < 0.0001); pairwise comparisons significant by Dunn’s post‐test are indicated by asterisks. The number of samples in each dataset is indicated on the graphs. **(c)** Microarray data from the TCGA GBM dataset were used to perform Kaplan–Meier survival analysis comparing overall survival of patients whose tumors were in the top 10% (*FAP*‐high) or bottom 10% (*FAP*‐low) of the expression range for *FAP* (*n* = 53 for each). Patients with *FAP‐*high tumors had significantly poorer survival. **(d)** *FAP* gene expression values, measured by RNAseq, were obtained from the GTEx portal for 51 normal tissue types and compared to cultured skin fibroblasts (black arrow; positive control). Box plots show median and 25th and 75th percentile; points are displayed as outliers if they are above or below 1.5 times the interquartile range. Number of samples analysed per tissue type ranged from 4 to 803, with a mean of 325. Blue dotted arrow highlights the 13 regions of brain tissue analysed.

To examine *FAP* gene expression in large patient cohorts, we mined published microarray and RNA sequencing datasets. Microarray data from The Cancer Genome Atlas (TCGA) revealed a significant overexpression of *FAP* in glioblastoma compared to normal brain (Figure 1a). By setting a conservative threshold for expression based on the mean + 3 × SD of the normal tissue samples, 39.6% of glioblastoma tissues (216/548 specimens) expressed *FAP* above the threshold, whereas none (0/9) of the normal brain tissues did. To support these microarray‐based analyses, we also analysed RNA sequencing data from TCGA (Figure 1b). This revealed that both primary and recurrent glioblastoma expressed *FAP* at significantly higher levels compared to less aggressive low‐grade gliomas, with no significant difference in expression between primary and recurrent tumors.

The above analyses revealed that some glioblastoma tissues show particularly elevated *FAP* expression. To determine whether such marked overexpression was associated with poorer prognosis, we compared survival time for patients in the top 10% (‘*FAP*‐high’) and bottom 10% (‘*FAP*‐low’) of the *FAP* expression range for the microarray dataset (Figure 1c). Indeed, the *FAP‐*high patients had significantly worse survival compared to the *FAP‐*low patients (*P* < 0.05). As observed in a previous analysis of TCGA data,^25^ *FAP* expression was particularly enriched in the mesenchymal tumors (Supplementary figure 1), in keeping with the poor prognosis of this subtype.^27^, ^28^ Interestingly, though, this previous analysis did not detect the association between *FAP* expression level and overall survival that we did, likely because samples were stratified into quartiles rather than comparing the top and bottom 10% of the expression range. Supplementary figure 1 also shows that high *FAP* expression was associated with overexpression of gene signatures for (1) vascular function; (2) immune system; and (3) extracellular matrix remodelling and interactions. The link with vascular genes is particularly interesting in light of other findings to be discussed below.

To avoid off‐tumor toxicity, an ideal immunotherapy target antigen shows low to negligible expression in healthy tissues. Previous studies suggest that FAP meets this criterion,^17^, ^18^, ^20^, ^21^, ^22^ but other studies have countervailing data.^23^, ^29^ To help clarify this issue, we examined *FAP* expression in a broad range of normal tissues by analysing the GTEx dataset, which includes RNAseq data from 53 different tissue types collected from healthy individuals, as well as cultured skin fibroblasts (Figure 1d). In agreement with results in Figure 1b, and the findings of others,^20^, ^22^ *FAP* transcripts were essentially undetectable in all 13 brain regions examined (median 0.31 transcripts per million (TPM) averaged across the different regions for all individuals). In contrast, cultured skin fibroblasts, which would be expected to express high levels of *FAP*, had a median TPM value of 111.3. Most other (unmanipulated) normal tissues had low levels of *FAP* expression, although certain tissues did show marginally elevated expression, particularly uterus (median 34.7), cervix (median 15.5 and 15.0 for endocervix and ectocervix, respectively) and arteries (median 23.5, 17.1 and 14.0 for aorta, coronary and tibial arteries, respectively).

Together, these analyses reveal that *FAP* gene expression is elevated in glioblastoma compared to either normal brain or lower grade gliomas, with high levels of expression associated with poor survival. Furthermore, *FAP* expression is negligible to low in all normal tissues examined, including normal brain. These expression patterns suggest FAP as a valuable immunotherapy target antigen unlikely to elicit on‐target/off‐tumor toxicity.

### Broad expression of FAP in glioblastoma tissue confirmed by immunohistochemistry (IHC)

**Figure 2:**
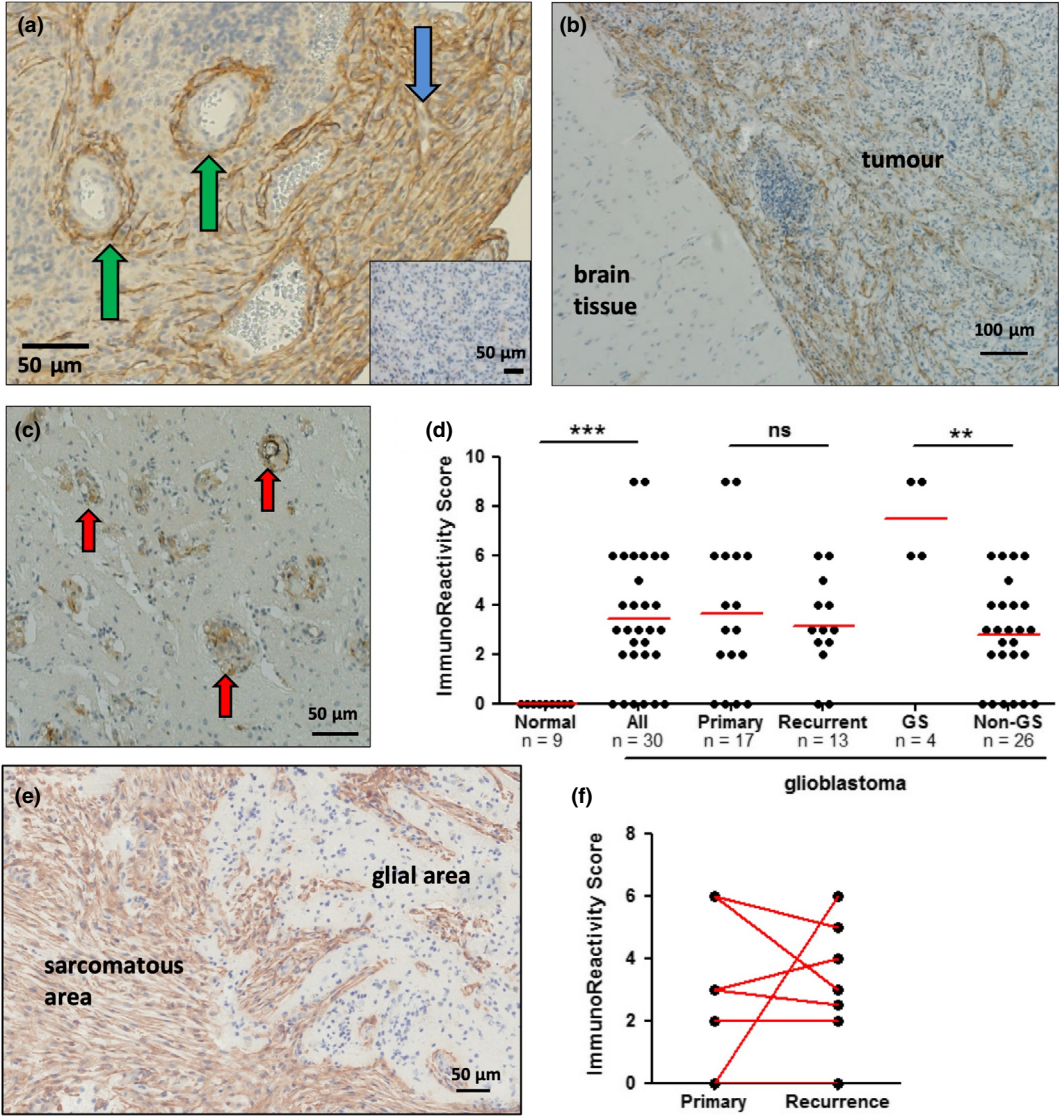
Immunohistochemistry confirms broad expression of FAP in glioblastoma tumor tissue: Glioblastoma tissue sections were stained to detect expression of FAP (brown), with nuclei counterstained with haematoxylin (blue). **(a)** An example of staining for FAP is shown, compared to staining with a control antibody (inset). The tissue stained for FAP highlights the two distinct patterns commonly observed: perivascular staining around, and sometimes within, vessel walls (green arrows); and fibrillary staining of the main tumor parenchyma (blue arrow). **(b, c)** Examples of two different types of tumor margin, showing distinct differences in FAP expression between normal and tumor tissue. The example in **b** shows a clearly demarcated border, while that in **c** shows FAP^+^ tumor cell nests invading normal tissue (red arrows). **(d)** A summary of FAP staining within the main tumor parenchyma for different types of tumors, compared to adjacent normal tissue. Differences in the group analysis were significant by 1‐way ANOVA; asterisks indicate significance by Dunn’s post‐test. The number of samples in each group is indicated in the graph. **(e)** Example of FAP staining in a gliosarcoma specimen, with areas of sarcomatous and glial morphology indicated. **(f)** Comparison of FAP expression in matched pairs of primary and recurrent tumors for 8 patients.

To assess FAP expression at the protein level, we performed IHC on 30 formalin‐fixed paraffin‐embedded (FFPE) specimens from histologically confirmed glioblastoma tumors (Supplementary table 1) using affinity‐purified sheep antiserum.^30^ FAP expression within these tumors was observed in two distinct patterns, as illustrated in Figure 2a, with staining of the main tumor parenchyma highlighted with the blue arrow and (peri)‐vascular staining highlighted with green arrows.

Nine of the biopsy specimens included an adjacent area of nontumor tissue, and examination of these areas revealed that they uniformly lacked FAP expression. Figure 2b shows a representative example of the interface between tumor and nontumor tissue, demonstrating that FAP expression was entirely limited to the tumor area. A second example is shown in Figure 2c, in which small clusters of FAP^+^ glioblastoma cells were observed invading the tumor‐adjacent brain tissue lacking FAP expression (red arrows). Further studies revealed that FAP expression was also completely lacking from healthy brain tissue in a total of 12 regions from 6 tissue donors who died from non‐neurological causes (Supplementary table 2).

To quantify FAP expression in glioblastoma, each tumor biopsy specimen was assessed for the breadth and intensity of FAP staining within the main tumor parenchyma, and adjacent nontumor tissue where available, using the ImmunoReactivity Score^31^, ^32^ (Figure 2d and Supplementary table 1). This analysis revealed that 14/16 primary tumors (87.5%) and 11/13 recurrent tumors (84.6%) displayed some level of FAP staining within the main tumor parenchyma, while 0/9 adjacent nontumor areas (0%) stained positive. Of interest, four cases of the gliosarcoma variant were included in our analysis, and all of these had particularly strong expression of FAP (*P* < 0.005 compared to nongliosarcoma tumors). Within gliosarcomas, FAP staining was virtually uniform in the portion of the tumor showing sarcomatous, spindle morphology (mesenchymal‐like) but absent from the glial cell compartment (Figure 2e). There was no significant difference in FAP staining between primary and recurrent glioblastomas as a group (Figure 2d). Furthermore, when matched primary‐recurrence pairs were compared for 8 patients, the level of FAP staining was not significantly different by a paired *t*‐test (Figure 2f). Primary tumors that expressed FAP maintained expression at recurrence in all cases, although the breadth and/or intensity sometimes varied. Three primary tumors lacked FAP expression; one gained expression at recurrence while the other two still lacked expression in the recurrent tumor. Quantification of the vascular staining pattern observed by IHC analysis was difficult without a definitive way to identify every vessel. This is addressed later in the study using 2‐colour immunofluorescence staining.

In summary, this IHC study confirms that FAP is broadly expressed in glioblastoma tissues but not at all in healthy brain. Furthermore, our data suggest that FAP is particularly prevalent in the gliosarcoma variant and is relatively stable between primary and recurrent tumors. Finally, we confirm the findings of Busek *et al*.^25^ that FAP is expressed in both the main tumor parenchyma and the vascular/perivascular niche, a concept that is explored in detail below.

### Patient‐derived short‐term cultures of glioblastoma cells express cell surface FAP, whereas cultured normal neurons and astrocytes do not

**Figure 3:**
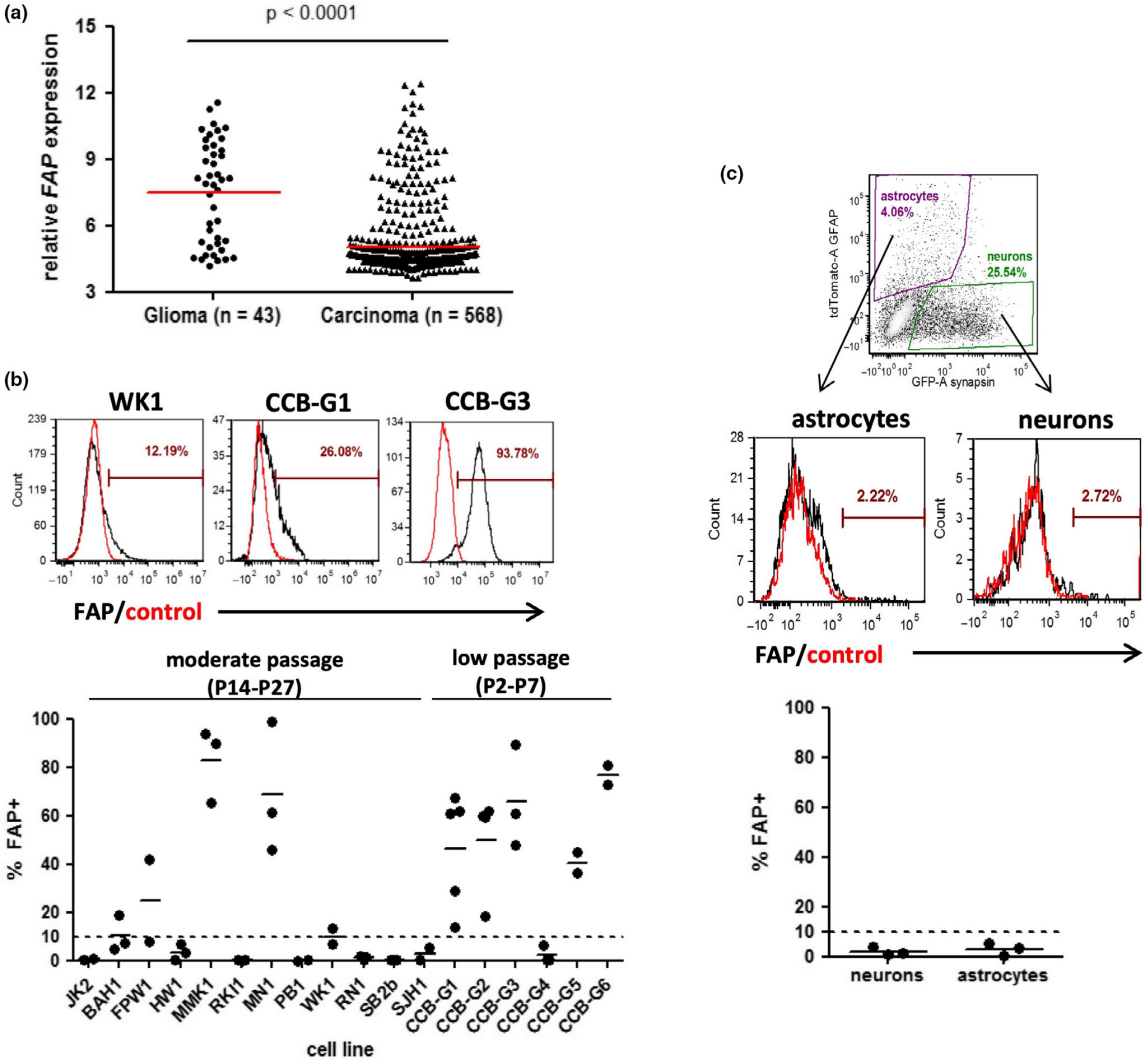
Expression of FAP by cultured glioblastoma cells but not neurons or astrocytes. **(a)** *FAP* gene expression values were extracted from the Cancer Cell Line Encyclopedia (CCLE) microarray dataset (GSE36133) using R. Cell lines derived from glioma (*n* = 43) or various carcinomas (*n* = 568; including lung, breast, colorectal, ovarian, pancreatic and others) were compared using box‐and‐whiskers plots spanning the interquartile range, with whiskers representing the 10^th^ and 90^th^ percentile and outliers shown as dots. Variation between groups was significant by the Mann–Whitney *U*‐test. **(b)** GNS cell cultures were assessed for expression of FAP by flow cytometry. The figure shows examples of FAP^+^ cultures containing a low, intermediate or high proportion of positive cells, with a summary of the percentage of cells staining positive for FAP within each culture shown below. The dotted line at 10% indicates the threshold above which expression was considered significant. Each culture was analysed multiple (2–5) times, with independent results indicated by dots and the mean value by a line. **(c)** Human embryonic stem (ES) cells were differentiated to generate mixed short‐term cultures containing both astrocytes and neurons, identified via lentiviral reporter expression of GFAP or synapsin, respectively. Flow cytometry was used to gate on viable cells (DRAQ5 + FVS575‐), and subsequently to gate the populations of interest on the basis of the reporter genes (top panel). FAP expression was then assessed for each, compared to isotype control (middle panels). The graph below shows a summary of 3 independent cultures.

To directly demonstrate FAP expression on pure populations of glioblastoma cells, we analysed a variety of cultured human cell types. Initially, microarray data from the Cancer Cell Line Encyclopedia (CCLE)^33^ were interrogated for expression of *FAP* in two collections of cell lines: those designated as ‘glioma’ (*n* = 43) and, for comparison, those designated as any type of carcinoma (*n* = 568) (Figure 3a). *FAP* was expressed at significantly higher levels in glioma lines than in carcinoma lines (*P* < 0.0001), in keeping with previous studies showing that FAP expression in carcinomas is limited to the cancer‐associated fibroblasts, rather than the tumor cells themselves.^18^

The cell lines analysed in the CCLE dataset are commercial lines that have been cultured for an undefined number of passages over many years or decades in serum‐containing medium, and therefore may not accurately represent the biology of glioblastoma cells *in vivo*. We therefore also assessed FAP expression on a panel of 18 glioma neural stem (GNS) cell cultures, using flow cytometry (Figure 3b). These cultures were expanded from patient glioblastoma tissue for a limited number of passages under serum‐free conditions that maintain an *in vivo*‐like cell phenotype.^34^, ^35^ This analysis revealed that GNS cell cultures frequently express cell surface FAP, ranging from a small subpopulation to uniform expression on every cell. Expression tended to be more common amongst very short‐term cultured cells (2–7 passages) compared to those that had been in culture longer (14–27 passages) (Figure 3b), with substantial FAP expression (>10% positive) observed for the majority (5/6) of short‐term cultures but only 5/12 longer‐term cultures. This could suggest that extended culture of GNS cells may result in gradual down‐regulation of FAP expression, although this possibility would require further longitudinal analysis to confirm.

For comparison, we also examined FAP surface protein expression in human stem cell‐derived neurons and astrocytes using flow cytometry. Neuronal cultures were matured for 1–3 months in BrainPhys neuronal medium to reach electrophysiological maturity as described previously.^36^, ^37^ The resulting cultures contained both neurons and glia, which were identified using lentiviral reporters expressing fluorescent proteins (GFP, tdTomato) under the control a neuronal promoter (synapsin 1) or astrocytic promoter (GFAP), respectively (Figure 3c). In three independent experiments, FAP expression was undetectable on both the neuron and astrocyte populations. These data are in agreement with the results reported in Figures 1 and 2, further supporting the concept that FAP expression does not extend to critical normal cell types found in the brain.

### Near‐ubiquitous expression of FAP by blood vessels in glioblastoma but not normal tissues

**Figure 4:**
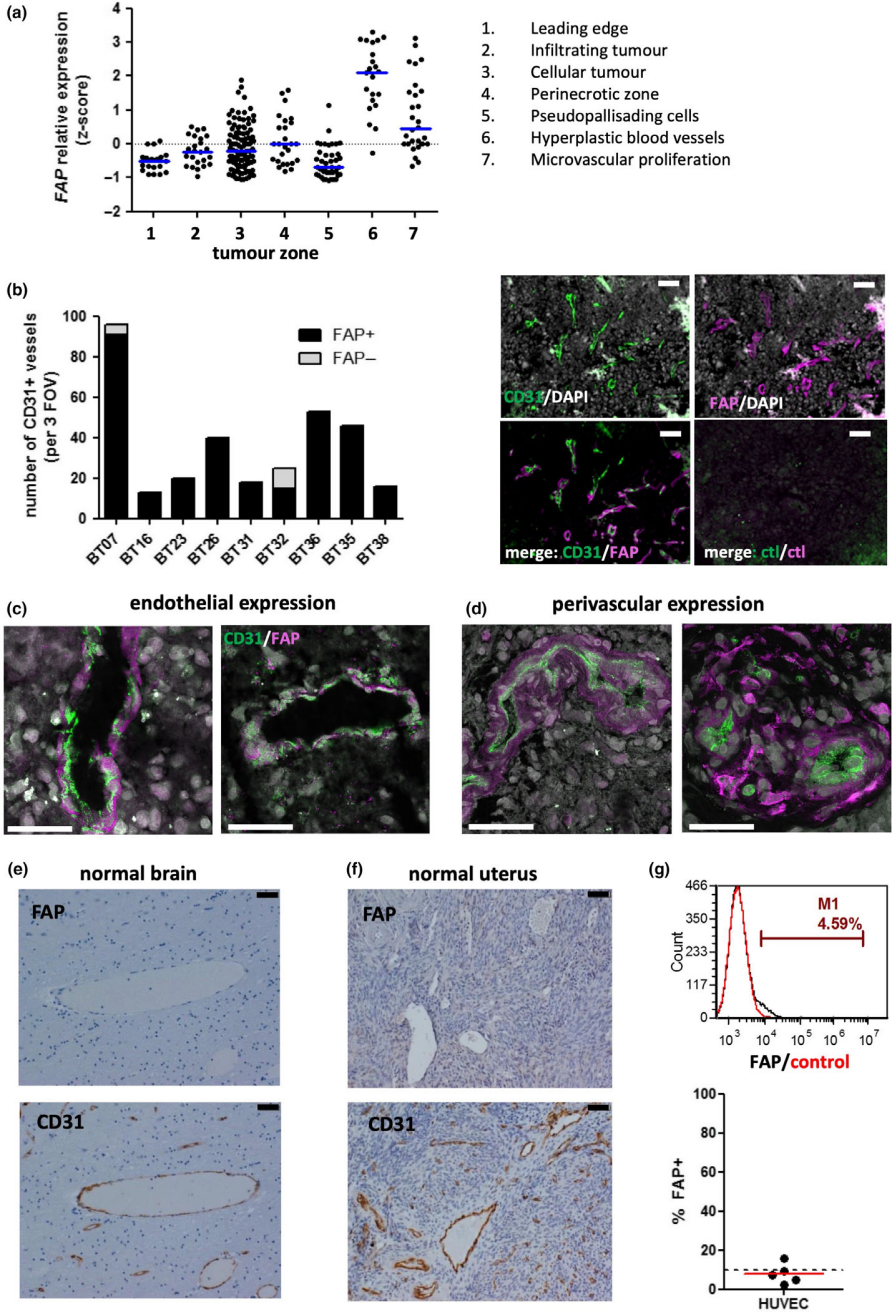
FAP is almost ubiquitously expressed by ECs and pericytes in glioblastoma, but not normal tissues. **(a)** RNAseq data from the Ivy Glioblastoma Project^38^ were used to assess *FAP* expression within the indicated regions of glioblastoma tissues. The scatterplot shows normalised z‐score values for each patient specimen. **(b)** Epifluorescence microscopy images were collected using a 20X objective and used to quantify the proportion of tumor vessels (identified as CD31^+^; green) which also stained positive for FAP (magenta). A summary of the 9 specimens examined is shown on the left, representing the total number of vessel structures identified in 3 fields of view, while representative images of BT35 are shown on the right. Scale bar = 50 µm. **(c, d)** Confocal microscopy was used to determine colocalisation patterns of FAP and CD31 within **c** and around **d** vascular structures. Scale bar = 50 µm. **(e, f)** Normal brain **(e)** or cervix/uterus **(f)** tissues were stained using IHC for either FAP or CD31, as indicated. Example brain tissue shown in **e** is representative of a total of 18 tissue blocks examined, while example uterine tissue shown in **f** is representative of 3 specimens. Scale bar = 50 µm. **(g)** Short‐term cultured HUVEC was assessed for FAP expression by flow cytometry. A representative example histogram is shown above, while a summary of 5 independent cultures is shown in the graph below. The dotted line at 10% indicates the threshold above which expression was considered significant, as for GNS cells in Figure 3.

Our IHC studies suggested that FAP could be expressed not only by glioblastoma cells themselves, but also by cells associated with tumor vessels. In fact, in many biopsies, the vessel‐associated expression of FAP was more prominent than in the main tumor parenchyma. To further assess FAP expression in distinct areas of the glioblastoma microenvironment, we analysed the Ivy Glioblastoma Atlas transcriptomic dataset.^38^ Here, laser capture microdissection was used to sample cells from seven different histologically defined zones of patient glioblastoma tissues, with subsequent transcriptomic analysis of each zone performed using RNAseq. As shown in Figure 4a, expression of *FAP* was most striking in regions annotated as ‘hyperplastic blood vessels’ or ‘microvascular proliferation’, although expression in the main tumor parenchyma (‘cellular tumor’) and at the interface between tumor and normal tissue (‘infiltrating tumor’) was also notable.

To unambiguously quantify the proportion of tumor vessels expressing FAP in our own tissue samples, we performed immunofluorescence staining for FAP together with the EC marker CD31 on fresh‐frozen glioblastoma tissues from nine patients. Figure 4b shows the number of CD31^+^ vessels identified across three fields of view per specimen, categorised according to whether FAP costaining was associated with the vessel (FAP^+^) or not (FAP^–^). For two of the specimens (BT07 and BT32), the majority of vessels were FAP^+^ (95% and 60%, respectively). And for the other seven specimens, every vessel structure identified costained with FAP. Therefore, FAP is almost ubiquitously expressed in the immediate vicinity of glioblastoma tumor vessels.

In addition to quantifying the proportion of FAP^+^ vessels, it was also of interest to determine whether FAP was expressed by ECs themselves, or by a perivascular population, or both. Glioblastoma tissues costained for CD31 and FAP were therefore also examined using high‐power confocal microscopy. For some vessels, we could identify clear expression of FAP on cells directly lining the vessel lumen (Figure 4c). As expected, these FAP^+^ cells that formed the inner lining of vessels also expressed CD31, thereby identifying them as ECs. In contrast, for some vessels, expression of FAP was distinct from the vessel wall, with the FAP^+^ cells surrounding CD31^+^ ECs in a perivascular pattern (Figure 4d).

The observation of FAP expression by ECs in glioblastoma raised the possibility that FAP is also expressed by blood vessels in other tissues. To address this, we first examined archival FFPE tissues from 6 normal brains by staining tissue sections with FAP or CD31 using IHC (Figure 4e and Supplementary table 2). Three brain regions were examined for each tissue donor, together encompassing a total of 12 regions, including those in which glioblastoma commonly arises (Supplementary table 2). Although both large and small vessels were readily identified in all specimens, none expressed detectable FAP.

In Figure 1d, we noted that the healthy tissue type with the highest level of *FAP* gene expression was the uterus. This tissue is highly vascularised, raising the possibility that the *FAP* transcripts detected in the GTEx study were contributed by blood vessels. We therefore also examined 3 FFPE specimens of healthy uterine or cervical tissue by IHC for FAP and CD31 (Figure 4f). Again, CD31^+^ vessels were abundant, but none expressed FAP. In contrast, scattered FAP^+^ cells with mesenchymal morphology were noted within the stromal tissue of one of the specimens (endometrial cavity/myometrium from a nonmenstruating 73 yo woman; not shown), presumably reflecting reactive tissue fibroblasts.

Finally, FAP expression was also investigated on short‐term cultures (3 passages) of human umbilical vein endothelial cells (HUVEC) from 5 healthy donors (Figure 4g). Flow cytometry revealed that expression of FAP was marginal to undetectable in each of these cultures.

Together, these studies suggest that FAP is not commonly expressed by blood vessels within healthy tissues or by normal ECs, but can be strongly and consistently induced on ECs within the glioblastoma microenvironment.

### Defining cell types within the glioblastoma microenvironment using single‐cell RNA sequencing

Our results thus far demonstrate that, within the glioblastoma microenvironment, FAP can be expressed by not just the tumor cells, but also EC lining tumor blood vessels and a population of cells closely associated with the vessels. The identity of this latter FAP^+^ perivascular population is presently unclear and could include classical pericytes, mesenchymal stem/stromal cells (MSC), perivascular macrophages or a subpopulation of tumor cells (possibly stem‐like cells) residing in the perivascular niche. To address this question, and to more definitively identify the cell types in the glioblastoma microenvironment expressing FAP, we used single‐cell RNA sequencing (scRNAseq) analysis of dissociated tumor tissue.

**Figure 5:**
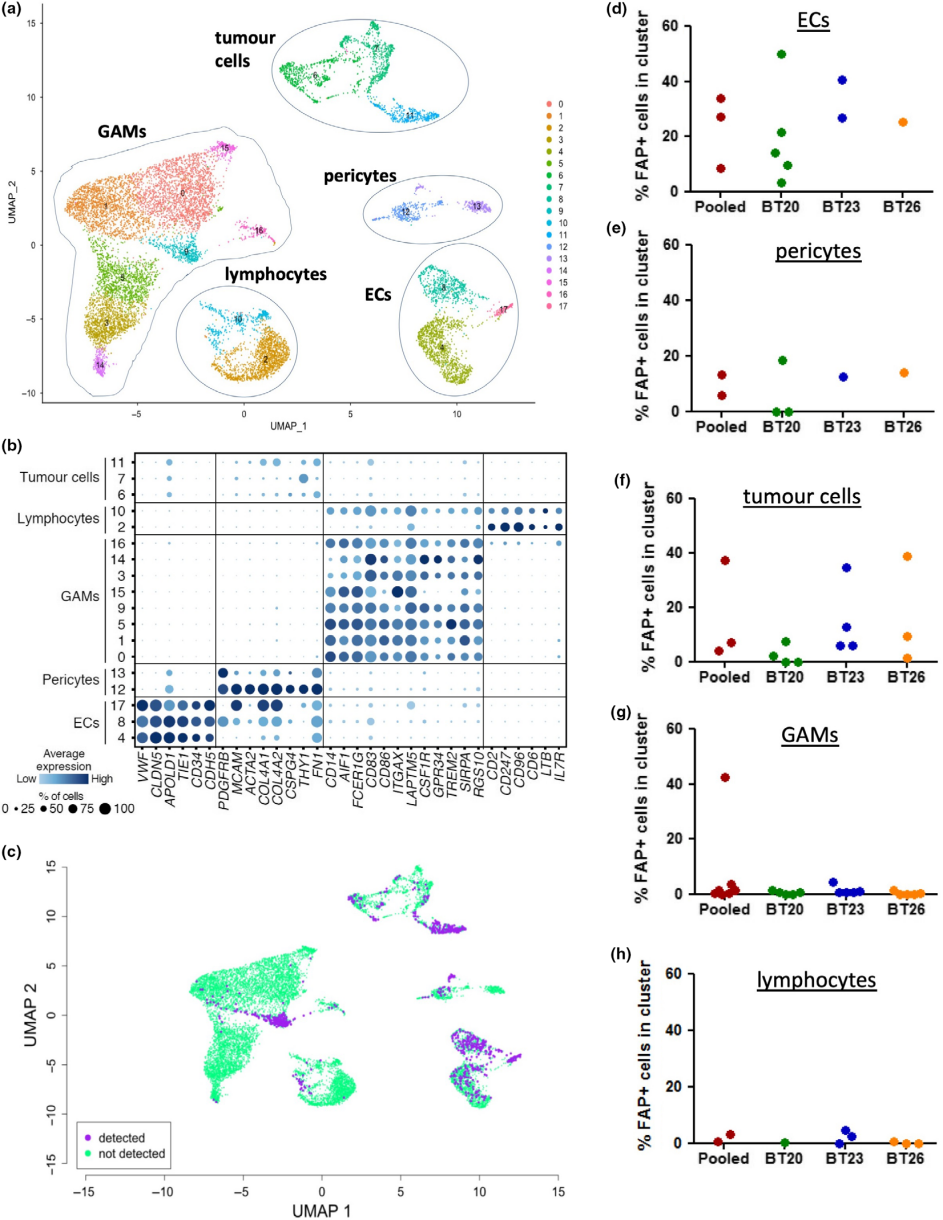
Single‐cell transcriptomic analysis of glioblastoma tissue specimens. Fresh tumor tissue specimens from three patients were dissociated to single‐cell suspensions and analysed by scRNAseq. **(** **a–c** **)** Results for the three patients pooled into a single dataset. **(d–h)** Summary graphs of the results for the pooled dataset and each individual specimen, as indicated. **(a)** UMAP plot showing unsupervised clustering of cells, with the cell type of each cluster annotated according to the presence of marker genes. **(b)** Dot plot showing the expression of marker genes used to define the indicated cell types within each numbered cluster. Size and colour intensity of dots indicates the percentage of cells in the cluster expressing the gene and the average level of expression, respectively. **(c)** The UMAP plot was coloured according to whether *FAP* transcripts were expressed (purple) or not (green) in individual cells. **(d–h)** Summary graphs showing the percentage of cells within indicated cluster types expressing detectable *FAP*. Each dot represents an individual cluster within the indicated dataset.

Tumor specimens were collected directly following surgery and dissociated to single‐cell suspensions using the gentleMACS system, followed by removal of myelin debris via magnetic bead purification. Cell suspensions were then processed using the 10X Chromium platform to generate transcriptomic data for individual cells within each of three patient tissue specimens. After filtering the datasets using standard quality control thresholds, a total of 4,668, 4,546 and 4,689 cells were available for analysis from the three specimens (BT20, BT23 and BT26), respectively. These datasets were pooled to give a total of 13,903 cells. Unsupervised clustering of the pooled dataset using principal component analysis was then performed and the resulting clusters of cells displayed on a UMAP plot (Figure 5a). The cell type identity of each cluster was deduced by searching for key lineage markers within lists of genes significantly overexpressed in that cluster compared to all other clusters. The breadth and level of expression of each of these marker genes within each cluster are summarised in Figure 5b.

Distinct populations of ECs, lymphocytes and glioma‐associated macrophages/microglia (GAMs) were identified within the pooled dataset (Figure 5a and b) and within each individual specimen (Supplementary figure 2). Each dataset also contained several clusters lacking stromal lineage markers but expressing a diverse range of genes associated with glial and neural cell types. We presume these clusters to constitute primarily tumor cells, but a detailed analysis falls outside the scope of the present study. The final cell type common to all three specimens was characterised by expression of *PDGFRB* (PDGFRβ/CD140b), *MCAM* (CD146), *THY1* (CD90), *FN* (fibronectin) and type IV collagen genes (Figure 5b), markers typical of mature pericytes, including those in normal human brain and glioblastoma.^39^, ^40^, ^41^ Although pericytes are notoriously difficult to define because of a lack of unique markers, the identification of at least one well‐demarcated cluster in each sample that expresses all of these pericyte‐associated genes strongly implicates this population as mature pericytes. Notably, none of the clusters designated as pericytes expressed significant levels of *NT5E* (CD73) or *ENG* (CD105), which are key markers of MSCs, a cell type with multilineage differentiation potential that expresses some overlapping markers with pericytes.^42^ Therefore, in contrast to a previous report,^43^ our data do not support the concept that MSCs are a significant component of the human glioblastoma microenvironment.

Hence, scRNAseq analysis has identified five key cell types present within the glioblastoma microenvironment: tumor cells, GAMs, lymphocytes, ECs and pericytes. To assess *FAP* gene expression within these different cell types, we coloured individual cells on the UMAP plots according to whether *FAP* transcripts were detected or not, for both the pooled dataset (Figure 5c) and individual specimens (Supplementary figure 2). In addition, a quantification of the percentage of cells expressing *FAP* in each cluster type is shown in Figure 5d‐h. *FAP* expression was negligible in lymphocyte and GAM clusters, with the exception of a single GAM cluster which also showed strong overexpression of gene pathways associated with response to cellular stress. In contrast, *FAP* was frequently detected within clusters identified as ECs, pericytes and tumor cells. The most consistent expression was in the EC clusters, with *FAP* expression in pericytes and tumor cell clusters being more variable. These findings are in concordance with our other results demonstrating expression of FAP by tumor cells and ECs and also suggest that at least some of the FAP^+^ cells localised around vessels are pericytes.

### High‐parameter flow cytometry confirms cell surface expression of FAP on ECs and pericytes

**Figure 6:**
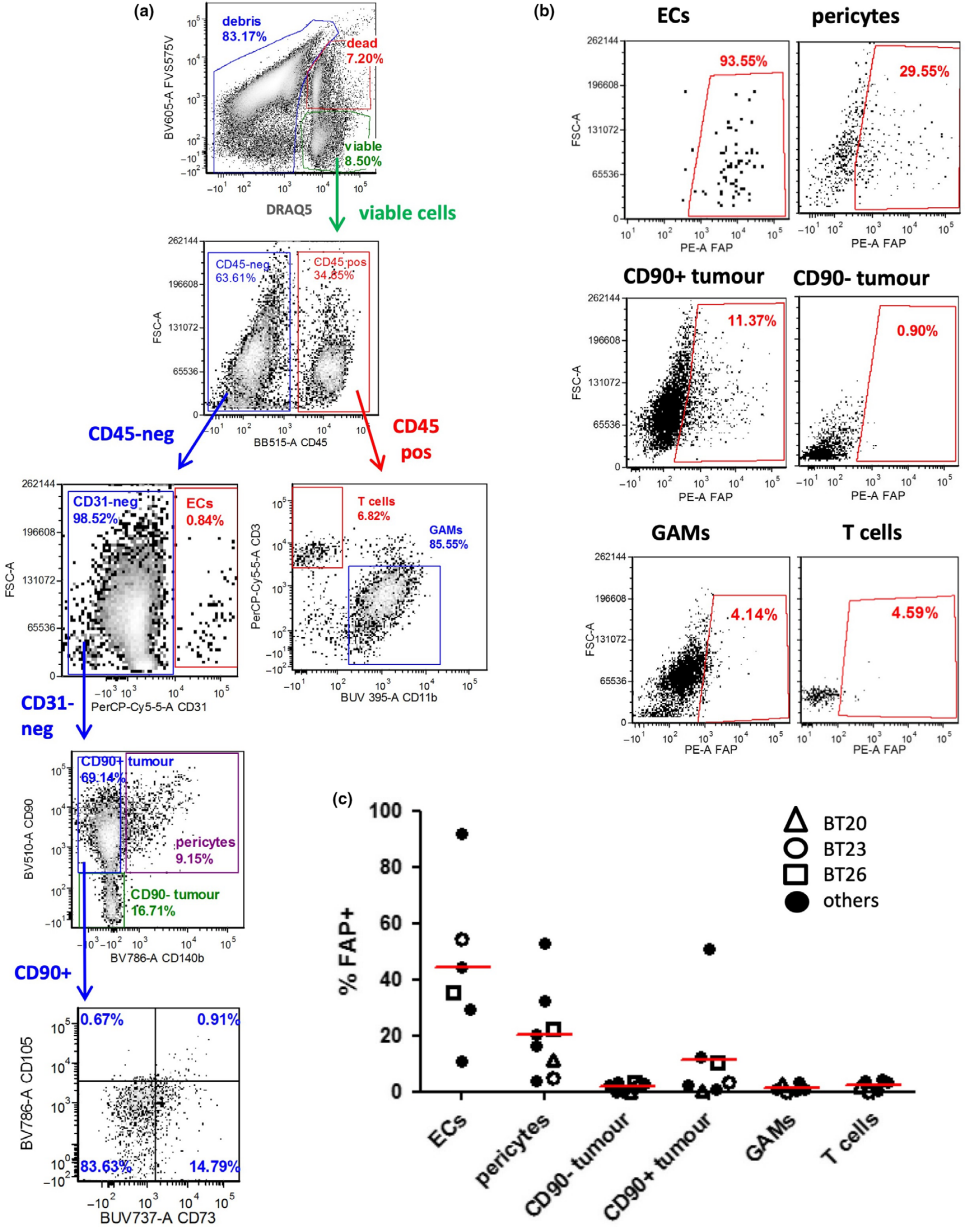
High‐dimensional flow cytometric analysis of dissociated tumor specimens confirms FAP expression by multiple cell types within tumors. Freshly isolated glioblastoma tumor specimens (*n* = 8) were dissociated to single‐cell suspensions and analysed using 9‐colour flow cytometry. **(a)** Representative example showing the flow cytometric gating strategy. **(b)** For each gated population of interest, expression of FAP was determined. Gates were set such that the per cent positive in the fluorescence minus one (FMO) control was < 1%. **(c)** Summary of FAP expression within the indicated cell populations for each specimen. Per cent positive values were determined by subtracting the FMO control value from the FAP‐stained value. Specimens matched to the scRNAseq analysis are highlighted with different shaped open symbols.

To obtain final proof that cell surface FAP is expressed by both ECs and pericytes in glioblastoma, we analysed dissociated glioblastoma tissue specimens (*n* = 8) using high‐parameter flow cytometry (Figure 6). The gating strategy used to identify the various cell types is outlined in Figure 6a. Viable cells were first discriminated from dead cells and noncellular debris according to positive staining with the nuclear dye DRAQ5 and lack of staining with the dead cell dye FVS575V. These viable cells were then identified as ECs (CD45^− ^CD31^+^), pericytes (CD45^−^ CD31^−^ CD90^+^ PDGFRβ^+^), GAMs (CD45^+^ CD11b^+^ CD3^−^) or T cells (CD45^+^ CD3^+^ CD11b^−^). The remaining population of cells lacking any stromal lineage markers could be clearly divided into CD90^+^ and CD90^−^ fractions, with the CD90^+^ fraction more abundant than the CD90^–^ fraction in 6/8 specimens (mean ratio CD90^+^/CD90^−^ = 5.27). To address whether some of these CD90^+^ cells could be MSCs, we assessed co‐expression of the other key markers of this cell type: CD73 and CD105.^42^ Expression of CD73 was variable, with 24.9 ± 19.8% (mean ± SD) co‐expressing this MSC marker, whereas CD105 expression was uniformly absent. In contrast, CD105 was readily detectable on ECs, as expected (not shown). The absence of a distinct population of cells co‐expressing CD90, CD105 and CD73 is in keeping with our scRNAseq data and further supports the conclusion that MSCs are not a major component of the glioblastoma microenvironment. Furthermore, we conclude that the most likely identity of the CD45^–^ CD31^–^ PDGFRβ^–^ population is tumor cells, with a subset expressing CD90 as previously described.^44^, ^45^, ^46^

The expression of FAP within each of the six identified cell types is shown in Figure 6b, c, with those specimens also analysed by scRNAseq highlighted with different shaped open symbols. FAP expression was most prominent amongst ECs and pericytes, with lower expression on CD90^+^ tumor cells, and no detectable expression on CD90^–^ tumor cells, GAMs or T cells. In general, the frequency of FAP^+^ tumor cells detected by flow cytometry was lower than that detected by scRNAseq, especially for specimen BT26. Also, for specimen BT23, *FAP* transcripts were detected within some of the GAMs by scRNAseq but these cells completely lacked detectable FAP in the flow cytometry analysis. Aside from these exceptions, the results of the flow cytometry analysis were generally in accordance with the scRNAseq analysis.

### FAP mRNA is also overexpressed in paediatric brain tumors

**Figure 7:**
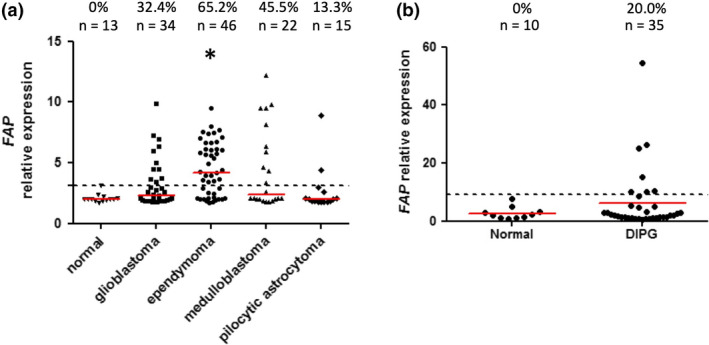
Transcriptomic analysis of FAP expression in paediatric brain tumors. *FAP* gene expression values were extracted from microarray dataset GSE50161 **(a)** or GSE50021 **(b)**. The relative expression value for each tissue sample is shown. Red lines represent the median of each group, while dotted lines represent the threshold for FAP expression, based on [mean + (3 x SD)] of the normal brain dataset. The total number of samples in each group, and the proportion with expression above the threshold, is indicated at the top of the graphs. **(a)** All groups were compared using the Kruskal–Wallis test (*P* < 0.005); pairwise comparisons significant by Dunn’s post‐test compared to normal brain are indicated by asterisks. **(b)** Comparison of DIPG vs normal using the Mann–Whitney *U*‐test was not significant.

All analyses conducted so far have focussed on glioblastoma in adults, but glioblastoma also occurs in the paediatric population. In addition, children are affected by several other forms of glioma which are rare in the adult population.^47^ Analysis of two published microarray datasets (GSE50161 ^48^ and GSE50021 ^49^) revealed that *FAP* gene expression was above normal brain levels in a variable proportion of tumor tissues for five distinct paediatric glioma types (Figure 7). For paediatric glioblastoma, the proportion of FAP‐expressing tumors (32.4%) was comparable to the adult disease, while pilocytic astrocytoma, a lower grade glioma, was characterised by a lower frequency of *FAP‐*expressing tumors (13.3%), again similar to adult low‐grade glioma. The frequency of *FAP‐*expressing tumors was highest for ependymoma, with 65.2% of tumors having *FAP* expression levels above the threshold. Medulloblastoma was also characterised by a relatively high frequency of *FAP*‐expressing tumors (45.5%). A small subset (20%) of diffuse intrinsic pontine glioma (DIPG) tumors also showed elevated *FAP* expression. These data support the concept that paediatric brain tumors could also potentially be targeted by FAP‐directed immunotherapy approaches, although this would require further validation at the level of protein expression.

## Discussion

In this study, we reveal FAP to be a potentially useful target antigen for novel glioblastoma immunotherapies. FAP was expressed at the gene and protein level by a subset of glioblastoma tumor cells, and more broadly by the tumor vasculature. Gene expression data also suggested overexpression of *FAP* in several paediatric brain tumor types. In contrast, normal tissues and healthy blood vessels largely lacked FAP expression. Of note, FAP is already being pursued as an immunotherapy target for various carcinomas, using approaches such as CAR‐T cells,^14^, ^15^, ^17^, ^50^, ^51^ immunotoxins,^13^, ^52^ radioimmunotherapy,^53^, ^54^ bispecific T‐cell engagers^16^ and nanoparticle‐based photoimmunotherapy.^55^ This interest is driven by the well‐established expression of FAP by stromal fibroblasts within the microenvironment of epithelial cancers, despite limited expression by the tumor cells themselves.^17^, ^18^, ^19^, ^56^, ^57^ Our data suggest that such therapeutic strategies could be extended to glioblastoma, where a locoregional route of administration may not only improve therapeutic results^58^, ^59^ but also take advantage of the identified patterns of FAP expression, which are limited to tumor cells and supporting vascular networks.

Several lines of evidence in our study demonstrate that FAP is expressed by a subset of human glioblastoma tumor cells. Tumor cell expression of FAP was observed by means of (1) IHC analysis of patient glioblastoma tissues; (2) flow cytometric analysis of GNS cells; (3) gene expression studies of long term cell lines; (4) single‐cell transcriptomics; and (5) flow cytometric analysis of dissociated tumor tissue specimens. Of all the approaches used, analysis of dissociated tumor tissue by flow cytometry revealed the lowest levels of FAP on tumor cells, with only a small fraction showing detectable expression. This could be because of the cleavage of a portion of the FAP protein molecules from the cell surface during the enzymatic tissue dissociation process. However, the use of a GentleMACS enzyme kit specifically designed to retain surface epitope expression and the ready detection of FAP on ECs and pericytes by flow cytometry argue against this possibility. Alternatively, any tissue dissociation process, especially where downstream purification steps are employed, has the potential for selective loss of certain cell types, but this is offset by the benefits of being able to deeply interrogate individual cells. It is for reasons such as these that we have undertaken such an extensive analysis of FAP expression at the gene and protein level using a broad range of techniques.

Previous studies have also observed expression of FAP in glioblastoma cell lines and tissues, but the degree of intratumor heterogeneity has not been well defined.^22^, ^25^, ^26^ It has therefore remained unclear what proportion of tumor cells would be targeted by FAP‐directed immunotherapies. Immunostaining of tissue sections revealed that, although most patient specimens expressed some level of FAP on the tumor cells, the intensity of expression and the proportion of cells positive varied widely, with particularly strong expression noted on the gliosarcoma variant. In gliosarcoma, FAP expression was limited to the mesenchymal/sarcomatous areas of these biphasic tumors, being absent from the glial areas. We have also recently observed an analogous expression pattern for two other glioblastoma antigens, EphA3 and α‐dystroglycan, in gliosarcoma.^60^ Intriguingly, these two molecules also show a perivascular distribution in glioblastoma and their expression is associated with the mesenchymal state,^34^, ^60^ suggesting that FAP, EphA3 and α‐dystroglycan could be functionally linked. We also observed that FAP expression was just as prominent on recurrent tumors as compared to primary tumors. This was important to establish, considering that future FAP‐targeting immunotherapies for glioblastoma would likely be initially tested in the setting of recurrent disease following failure of standard therapies.

Whether the FAP^+^ tumor cells have a unique function, or represent a particular differentiation state, in comparison with the FAP‐negative fraction is presently unclear. Interestingly, flow cytometric analysis of dissociated tumor tissue revealed that almost all tumor cells in which FAP was detected also co‐expressed the CD90 (Thy‐1) surface antigen. CD90 has been suggested as a marker of glioma stem cells (GSC),^45^, ^46^ raising the possibility that FAP could be preferentially expressed on this subset. More recently, CD90 has been shown to drive glioblastoma cell invasion,^44^ suggesting that FAP might mark a particularly invasive subset of tumor cells. This is in keeping with its function as a protease capable of degrading extracellular matrix proteins,^61^ and our observation that clusters of tumor cells invading tumor‐adjacent brain tissue expressed FAP. This concept is also supported by our finding that overexpression of *FAP* was associated with poor prognosis. Thus, FAP may be a particularly relevant immunotherapy target antigen, despite its heterogeneity of expression, because it would allow targeting of the invasive and/or stem‐like cells responsible for continued tumor growth and disease recurrence after standard treatments.^12^

In comparison with the heterogeneity of expression observed on tumor cells, expression of FAP in the vascular compartment of glioblastoma was remarkably consistent. In fact, for most tumors examined, every CD31^+^ blood vessel was associated with strong FAP staining by immunofluorescence. Furthermore, analyses of the Ivy Glioblastoma dataset revealed intense expression of *FAP* in areas of microvascular proliferation and hyperplastic blood vessels selected by laser capture microdissection. Vascular‐localised expression of FAP has also been previously noted in breast cancer^62^ and multiple myeloma.^63^ In contrast, blood vessels in normal breast tissue lacked FAP expression,^62^ as did healthy human aortas.^64^ These latter observations are in keeping with our IHC results, which revealed that CD31^+^ vessels in normal brain, uterus and cervix lacked FAP expression. Therefore, expression of FAP appears to be a unique feature of angiogenic tumor blood vessels, compared to healthy vessels, and is particularly prominent in glioblastoma.

Vessel‐localised expression of FAP in glioblastoma has been observed previously, but the identity of the FAP^+^ cells within this niche was not clear.^25^ This previous study demonstrated that, in tumors displaying epidermal growth factor receptor (EGFR) amplification, the FAP^+^ perivascular population lacked EGFR amplification, thereby identifying them as a stromal cell type rather than a subtype of tumor cells residing in the perivascular niche. These cells also had high expression of fibronectin and smooth muscle actin, suggesting they could represent pericytes, MSCs or fibroblasts. We now reveal, using a combination of flow cytometry and single‐cell transcriptomics, that a large proportion of the FAP^+^ stromal cells have a phenotype consistent with classical pericytes. This includes expression of PDGFR‐β/CD140b, MCAM/CD146, Thy‐1/CD90, fibronectin and several collagen genes, all of which have emerged as reliable markers of human pericytes in normal brain and glioblastoma.^39^, ^40^, ^65^ We have confirmed that these cells do not represent MSCs, as they lacked expression of the key MSC markers CD73 and CD105.^42^ Of note, FAP expression has also been documented on pericytes in breast cancer,^56^ and FAP has been investigated as a target for pericyte‐directed vascular disrupting agent (VDA) therapy in several tumor models.^66^ Thus, FAP expression may be a common feature of tumor pericytes.

In addition to pericytes, we observed distinct expression of FAP on ECs. This is in contrast to the study by Busek *et al*., who did not detect expression of an EC marker (vWF) by FAP‐expressing cells associated with tumor vessels.^25^ However, we are confident in our conclusion, based on our observations that (1) single‐cell transcriptomics demonstrated expression of *FAP* by cells within the EC cluster; (2) flow cytometry confirmed expression of FAP surface protein by a large proportion of cells with a CD45^−^ CD31^+^ phenotype; and (3) using confocal microscopy, FAP and CD31 co‐expression was observed immediately adjacent to vessel lumens.

In addition to pericytes and ECs, it is also possible that a fraction of the perivascular‐localised FAP is because of expression on GSCs, which preferentially reside in the perivascular niche.^67^ Furthermore, some of the perivascular FAP^+^ cells observed in tissue sections had a nuclear morphology more resembling tumor cells than pericytes, and sometimes were located a considerable distance away from the vessel (e.g. see Figure 4d, right panel), arguing against a pericyte identity. Intriguingly, lineage‐tracing studies in mice, together with analyses of human tumor specimens, have demonstrated that GSCs can differentiate into tumor pericytes.^41^ Thus, the FAP^+^ cells clustered around vessels could represent a mix of GSCs and pericytes derived from them.

Blood vessels are considered a key therapeutic target in glioblastoma and many other cancers, based on the concept that starving a tumor of its blood supply should result in tumor shrinkage or even elimination. Indeed, the angiogenesis inhibitor bevacizumab (Avastin) has been approved by the United States Food and Drug Administration (FDA) for the treatment of glioblastoma, but clinical responses to this treatment are generally limited because of resistance mechanisms that develop within the tumor over time.^68^ We propose that FAP‐targeted immunotherapy could be a more effective approach to targeting the glioblastoma vasculature by facilitating the rapid and selective physical destruction of existing tumor blood vessels, including their supporting perivascular network, rather than aiming to block a pro‐angiogenic signalling pathway that tumors can evolve to survive without.

Ideally, an immunotherapy target antigen demonstrates no expression on any healthy cells or tissues, to avoid on‐target/off‐tumor toxicities. In previous studies, FAP has been reported to show negligible to marginal expression in a range of healthy human tissues, including normal brain.^17^, ^18^, ^20^, ^22^ In keeping with these observations, a radiolabelled anti‐FAP antibody (sibrotuzumab) showed highly selective uptake by tumors but not normal tissues in patients with colorectal carcinoma or non‐small‐cell lung cancer.^21^ In contrast, in mice, a reporter system was used to show that FAP^+^ stromal cells reside in almost every normal tissue,^23^ and analysis of murine multipotent bone marrow stromal cells revealed they were uniformly FAP^+^.^24^ To more comprehensively address the expression of *FAP* by a wide range of healthy human tissues, we interrogated the GTEx transcriptomic dataset. This analysis of hundreds of donors revealed that, of the 53 normal tissue types examined, *FAP* expression was predominantly restricted to major arteries (aorta, coronary and tibial) and the uterus and cervix, and even these tissues showed low expression compared to cultured fibroblasts. The low‐level *FAP* expression we observed in female reproductive organs is supported by a previous tissue microarray study, which revealed weak expression of FAP in cervix and uterus,^17^ and our own IHC studies, which detected scattered FAP^+^ cells in one sample. However, the marginal expression of the *FAP* gene that we detected in major arteries is in contrast to a previous study of atherosclerosis, which revealed that FAP was undetectable by immunofluorescence or Western blot in healthy human aortas.^64^ Thus, low‐level expression of the *FAP* gene may not necessarily lead to detectable protein expression. Together, our observations and those of others suggest that expression of FAP in healthy human tissues is limited to marginal levels on a small fraction of tissue types.

In keeping with the concept that FAP expression in healthy tissues is minimal, several different FAP‐targeting immunotherapies have shown very limited toxicity in mouse xenograft models, even when using anti‐FAP moieties that recognise both human and mouse FAP.^13^, ^14^, ^51^, ^52^ In contrast, one study reported significant bone marrow toxicity and lethal cachexia following injection of mice with FAP‐targeting CAR‐T cells.^24^ This observation may be related to the nature of the specific CAR structure used, because other studies using two alternative CAR structures reported a distinct lack of significant toxicities.^14^, ^51^ Moreover, a first‐in‐human clinical trial of FAP‐targeting CAR‐T cells has been launched in the setting of mesothelioma, with a first patient successfully infused.^15^ Thus, careful optimisation of the binding affinity of FAP‐targeting moieties is likely to allow for the development of new cancer immunotherapies with an excellent safety profile. Furthermore, in glioblastoma, local delivery of FAP‐targeting immunotherapies could further reduce the possibility of systemic toxicity, considering that FAP expression has never been observed in normal brain in previous studies^17^, ^20^, ^22^ or our own. Another aspect that should be considered in the development of FAP‐targeting immunotherapies is interaction with soluble FAP, a naturally occurring derivative of membrane‐bound FAP which is constitutively present in plasma and regulates fibrin clotting.^69^ Considering that soluble FAP is virtually identical to the extracellular portion of membrane‐bound FAP, this circulating protein could potentially block the antigen‐binding domain of FAP‐targeted therapeutics, although this possibility is yet to be formally explored. The levels of soluble FAP in the brain are unknown, but could well be lower than in plasma, making local delivery of FAP‐targeting therapies even more attractive.

Our study in human glioblastoma reveals heterogeneous expression of FAP on tumor cells, coupled with near‐ubiquitous expression around blood vessels, with both ECs and pericytes contributing to the observed vascular expression. The function of FAP on these diverse cell populations is presently unclear. Although we did observe that high *FAP* expression is associated with reduced overall survival in the TCGA glioblastoma dataset, this does not necessarily indicate a functional role for FAP in disease progression. Indeed, the relationship between *FAP* expression and reduced survival could be because of indirect associations, including a link with the mesenchymal phenotype, which has been shown to be clinically more aggressive,^27^, ^28^ and the expression of FAP by glioblastoma blood vessels, which are a negative prognostic factor when present at high density.^70^ However, previous studies have demonstrated a direct functional role for FAP in other cancer types,^71^ suggesting that this is an area worthy of future investigation in the context of glioblastoma. Nevertheless, it is important to note that it is the expression pattern *per se*, rather than biological function, which identifies a good target antigen for immunotherapy. Our detailed analyses of FAP expression, including evidence of surface protein expression (using flow cytometry and immunofluorescence staining of nonpermeabilised cells), suggest that it meets the criteria for an excellent immunotherapy target in glioblastoma. Indeed, our ongoing studies have demonstrated promising evidence of efficacy for FAP‐targeting CAR‐T cells in a mouse xenograft model of glioblastoma, even where intratumoral expression of FAP is heterogeneous, and a distinct lack of toxicity (manuscript in preparation). Thus, FAP‐targeting immunotherapies may be a promising therapeutic modality for an aggressive tumor type, the lethal natural history of which is only delayed by standard combination treatments.

## Methods

### Patient tissue samples and ethics

Patient tissue specimens were obtained through the South Australian Neurological Tumour Bank (SANTB), the South Australian Brain Bank or SA Pathology archival diagnostic specimens and used in accordance with the principles of the Declaration of Helsinki. Studies were approved by the Central Adelaide Local Health Network Human Research Ethics Committee (approval numbers RAH050310a, R20160727, R20160913 and R20140314), the Southern Adelaide Clinical Human Research Ethics Committee (approval numbers 286.10 and 051.045) and the Royal Brisbane and Women’s Hospital (HREC/17/QRBW/577).

Fresh glioblastoma tissue specimens from the SANTB were obtained as either pieces of resected tumor or as aspirates following tissue ablation by Cavitron Ultrasonic Surgical Aspirator (CUSA) and processed within 2 h of collection. For resected tumor pieces, any significant areas of necrosis or haemorrhage were discarded, and a portion of the remaining tissue embedded in Tissue‐Tek® O.C.T.™ medium (Sakura Finetek, Torrance, CA, USA) and immediately frozen in supercooled isopentane (Ajax Finechem, Taren Point, NSW, Australia) for subsequent cryosectioning. Remaining tissue was rinsed several times to remove visible blood and dissociated to generate a single‐cell suspension using the gentleMACS Octo Dissociator in combination with the Human Tumor Dissociation Kit (both Miltenyi Biotec Australia; Macquarie Park NSW), according to the manufacturer’s recommendations. For CUSA aspirates, tissue fragments were sedimented by slow (70 × *g*) centrifugation for 1 min and most of the liquid discarded. The remaining slurry of tissue fragments was dissociated using the gentleMACS system, as for resected tumor pieces. Following dissociation, cell suspensions were filtered through a 70‐µm cell strainer, any contaminating erythrocytes were lysed using ACK lysis buffer and the cells subjected to several washes in Dulbecco’s modified Eagle’s medium (DMEM; Sigma‐Aldrich Australia; North Ryde, NSW). Some cells were cryopreserved by resuspending in 90% foetal bovine serum (FBS; Thermo Fisher Scientific, Waltham, MA, USA) + 10% dimethyl sulphoxide (DMSO; Sigma‐Aldrich) and placing in a Mr Frosty freezing chamber (Thermo Fisher) at −80°C overnight, for future analyses, while remaining cells were used for generating GNS cell cultures (see below).

Formalin‐fixed paraffin‐embedded (FFPE) tissue biopsies were obtained from the archives of SA Pathology, the South Australian Brain Bank and the Royal Brisbane and Women’s Hospital, together with associated pathology reports. Human umbilical vein endothelial cells (HUVEC) were extracted from umbilical cords by collagenase digestion and cultured in HUVE medium on gelatin‐coated flasks, as previously described.^72^

### Generation and culture of Glioma Neural Stem (GNS) cell lines

The Q‐Cell GNS cell line series was generated and characterised as previously described.^73^, ^74^, ^75^ In addition, further cultures (the CCB‐G series) were generated de novo as follows: an aliquot of dissociated glioblastoma cells, prepared as described above, was resuspended in 3 mL StemPro NSC medium (Thermo Fisher) and transferred to a T‐25 tissue culture flask coated for 30 min at 37°C with Matrigel (Corning Incorporated, Corning, NY, USA) diluted 1/100 in PBS. The following day, the flask was rinsed twice with warm DMEM to remove debris and dead cells, and fresh StemPro NSC was added. All GNS cell cultures were passaged when they reached 70–95% confluence by detaching cells using StemPro Accutase (Thermo Fisher) and seeding fresh Matrigel‐coated flasks at a 1:3 to 1:5 split ratio. Cultures were grown in a humidified incubator at 37°C with 5% CO_2_.

### Generation of iPSC‐derived neurons and astrocytes and labelling with transcriptional reporters

WA09 (H9) human embryonic stem (hES) cells (WiCell, Madison, WI, USA) were differentiated into neural progenitor cells (NPCs) using an embryoid body (EB)‐based protocol described previously^36^, ^76^. NPCs were expanded on Matrigel‐coated 6‐well plates in neural progenitor medium (NPM), composed of DMEM/F12 + GlutaMAX™ basal medium (Thermo Fisher) supplemented with 1 × NeuroCult™ SM1 (STEMCELL Technologies, Vancouver, BC, Canada), 1 × N2 Supplement‐A (STEMCELL Technologies), 100 ng mL^−1^ FGF‐8b (PeproTech, Rocky Hill, NJ, USA), 200 ng mL^−1^ Sonic Hedgehog (PeproTech), 1 μg mL^−1^ laminin (Thermo Fisher) and 200 nM L‐ascorbic acid (Sigma‐Aldrich). NPCs were maintained at high density (2–4 × 10^5^ cells cm^−2^), fed every other day with fresh NPM and split about once a week onto fresh Matrigel using Accutase. For neuronal maturation, NPCs were dissociated and seeded at a density of 8 × 10^4^ cells cm^−2^ onto 48‐well tissue culture plates coated with 10 μg mL^−1^ poly‐L‐ornithine (Sigma‐Aldrich) and 5 μg mL^−1^ laminin (Thermo Fisher). Twenty‐four hours later, half the NPC medium was gently replaced with neuronal maturation medium (NMM): BrainPhys™ basal medium supplemented with 1 × SM1 and 1 × N2‐A (STEMCELL Technologies), 20 ng mL^−1^ BDNF (PeproTech), 20 ng mL^−1^ GDNF (PeproTech), 0.5 mM dibutyryl cyclic AMP (Sigma‐Aldrich), 200 nM ascorbic acid and 1 μg mL^−1^ laminin. Half of the neuronal medium was gently replaced three times a week. Plates were kept in a humidified incubator at 37°C with 5% CO_2_.

Transcriptional reporter lentivectors encoding EGFP and tdTomato fluorescent proteins were designed to identify neurons (expressing *Synapsin*) and astrocytes (expressing *GFAP*) in flow cytometry experiments of hES cell‐derived mixed neuronal cultures. Lentiviral vectors were produced in Lenti‐X™ 293T cells (Takara Bio, Kusatsu, Shiga, Japan) cultured in DMEM, high glucose, no glutamine (Thermo Fisher) supplemented with 10% FBS (Thermo Fisher), 4 mM GlutaMAX™ supplement (Thermo Fisher) and 1 mM sodium pyruvate (Sigma‐Aldrich) on tissue cultureware coated with 0.002% poly‐L‐lysine (Sigma‐Aldrich). Lenti‐X™ 293T cells were transfected with 12.2 µg lentiviral transfer vector (pCSC‐Synapsin‐MCS‐EGFP or pBOB‐GFAP‐tdTomato, both derivatives of pCSC‐SP‐PW‐EGFP^77^ and 3^rd^‐generation packaging plasmids (8.1 µg pMDL/RRE, 3.1 µg pRSV‐REV and 4.1 µg pCMV‐VSVg) using a polyethylenimine (PEI, 25 kDa, linear; Polysciences, Warrington, PA, USA) transfection method (4:1 ratio of PEI to DNA). The culture medium was exchanged six hours after transfection. The supernatants containing lentiviral particles were collected ~ 66 hours post‐transfection, filtered through a 0.45‐µm SFCA membrane filter and ultracentrifuged at 25,000 rpm (Beckman, Optima XPN) for 2 hours at 4˚C. Virus pellets were resuspended in Hank’s balanced salt solution (HBSS; Thermo Fisher), and virus titres were determined using the Lenti‐X™ qRT‐PCR Titration Kit (Takara Bio) following manufacturer’s instructions. Neuron cultures were simultaneously transduced with both reporter lentivectors (Synapsin‐EGFP: 2 × 10^3^ viral RNA copies/cell; GFAP‐tdTomato: 1 × 10^3^ viral RNA copies/cell) for 48 hours before media replacement with fresh NMM. Seven days after lentiviral transduction, the cells were harvested using Accutase, centrifuged and carefully resuspended in ice‐cold PBS for flow cytometric analysis.

### Immunohistochemistry staining of FFPE tissues

Formalin‐fixed paraffin‐embedded tissue blocks were sectioned, dewaxed and subjected to heat‐mediated antigen retrieval using a microwave (15 min once boiling point was achieved) in pH 8.0 Tris buffer (for FAP staining) or pH 6.5 citric acid buffer (for CD31 staining). After cooling, sections were washed and endogenous peroxidases were quenched using 1% hydrogen peroxide for 10 min. Nonspecific antibody binding was blocked using 10% normal rabbit serum (for FAP staining) or 10% normal goat serum (for CD31 staining), both from Vector Laboratories (Burlingame, CA, USA). Blocking solution was removed, and sections were incubated overnight at 4°C with sheep anti‐FAP (R&D Systems, Minneapolis, MN, USA), control sheep IgG (R&D Systems) or anti‐CD31 (Bethyl Laboratories, Montgomery, TX, USA). After extensive washing, sections were incubated with secondary antibody for 35 min at RT using rabbit anti‐sheep‐biotin (Sigma‐Aldrich) for FAP staining, or goat anti‐rabbit‐biotin (Vector Laboratories) for CD31 staining. After extensive washing, detection steps were performed using the Vectastain Elite ABC HRP kit (Vector Laboratories) according to the manufacturer’s recommendations, followed by reaction with DAB peroxidase substrate kit (Vector Laboratories). Sections were then counterstained using Mayer’s haematoxylin (Chem‐Supply, Port Adelaide, SA, Australia) and mounted in DPX mountant (Sigma‐Aldrich).

### Immunofluorescence staining of cryosections

Sections cut from OCT‐embedded fresh tissues (5–8 µm thickness) were fixed for 10 min in a 40:60 mix of methanol:acetone, washed in TBS and blocked using 10% normal donkey serum (Sigma‐Aldrich) in CAS Block (Thermo Fisher) at RT for 3 h. Primary antibodies were mixed together: sheep anti‐FAP (R&D Systems) plus mouse anti‐CD31 (clone 89C2: Cell Signalling Technology, Danvers, MA, USA), or control sheep IgG (R&D Systems) plus control mouse IgG1 (clone P3.6.2.8.1: eBioscience, San Diego, CA, USA) and incubated with tissue sections overnight at 4°C. After extensive washing, the secondary antibody cocktail containing AlexaFluor647‐conjugated donkey anti‐sheep IgG and AlexaFluor546‐conjugated donkey anti‐mouse IgG (Thermo Fisher) was added and incubated at RT for 2 h. After extensive washing, sections were mounted using ProLong Gold anti‐fade mounting medium with DAPI (Thermo Fisher) and allowed to cure overnight before viewing.

### Microscopy, imaging and scoring

Brightfield and epifluorescence images were captured using an IX73 microscope equipped with CoolLED pE‐4000 light source and running CellSens software (Olympus Australia, Notting Hill, VIC). Fluorescence overlays were created by overlaying black and white images and applying false colour using ImageJ. Confocal imaging was performed on an LSM 700 Axio Observer.Z1 confocal microscope equipped with 405 nm (5 mW), 488 nm (10 mW), 555 (10 mW) and 639 nm (5 mW) lasers, using a C Apo 40x/1.2 W DICII objective and Zen Black (v 8.1.5.484) software (Zeiss Australia, North Ryde, NSW). All fluorescence imaging was performed using consistent illumination, capture and display settings between samples. IHC staining was quantified using the ImmunoReactivity Score matrix,^31^, ^32^ whereby the main tumor parenchyma across the entire tissue section (excluding perivascular regions) was scored for the percentage of cells staining positive/breadth of staining (0 = none; 1 = 1–20%; 2 = 20–40%; 3 = 40–70%; 4 = >70%) and average staining intensity (1 = pale; 2 = mid; 3 = intense). These two values were multiplied to produce an overall score for each specimen. For some specimens, multiple tissue blocks were available for analysis, in which case the ImmunoReactivity Score was averaged across the samples.

### Flow cytometric staining and analysis

For staining of GNS cultures and HUVEC, cells were harvested using Accutase or TrypLE Select (Thermo Fisher), respectively, washed in PBS and resuspended in FACS buffer (PBS+ 1% bovine serum albumin (BSA) + 0.04% sodium azide). Cells were incubated with mouse anti‐FAP (clone 427819; R&D Systems), or with the same concentration of control IgG1 (eBioscience clone P3.6.2.8.1), for 20 min at RT. After washing, cells were incubated with goat anti‐mouse secondary antibody conjugated to either AlexaFluor488 or AlexaFluor647 (Thermo Fisher) for 20 min at RT, then washed in FACS buffer. In some experiments, the anti‐FAP mAb was directly conjugated to PE, in which case the incubation with secondary antibody was omitted.

For staining cultured neurons/astrocytes, cells were washed in PBS+ 1% BSA and incubated with mouse anti‐FAP (clone 427819; R&D Systems) or control IgG1 (eBioscience clone P3.6.2.8.1), for 20 min at 4°C. After washing in PBS+ 1% BSA, cells were incubated with anti‐mouse‐BV421 secondary antibody (Biolegend, San Diego, CA, USA) for 20 min at 4°C, washed in PBS and then incubated with a cocktail of fixable viability stain 575V (BD Biosciences Australia, North Ryde, NSW) and DRAQ5 (BD Biosciences) for 15 min at RT. After washing, cells were subject to flow cytometric analysis immediately. A positive control (cell line with known FAP expression) was run in parallel in each experiment to ensure that the method for FAP detection was functional.

For staining dissociated tumor specimens, cryopreserved samples were rapidly thawed in a 37°C water bath and transferred to tubes containing 9 mL warm RPMI + DNase I (Sigma‐Aldrich) at 20 U mL^−1^ to prevent cell clumping from free DNA. After 10 min at RT, cells were centrifuged and resuspended in FACS buffer, and Fc receptors were blocked by the addition of purified human IgG at 100 µg mL^−1^ for 10 min at RT. Cells were then incubated for 20 min at RT with cocktails containing Brilliant Staining Buffer Plus (BD Biosciences) and combinations of the following antibodies: anti‐FAP‐PE (clone 427819; R&D Systems), anti‐CD31‐PerCP‐Cy5.5 (clone WM‐59; Biolegend), anti‐CD3‐PerCPCy5.5 (clone UCHT1; BD Biosciences), anti‐CD45‐BB515 (clone HI30; BD Biosciences), anti‐CD90‐BV510 (clone 5E10; BD Biosciences), anti‐CD11b‐BUV395 (clone ICRF44; BD Biosciences), anti‐CD140b/PDGFRβ‐BV786 (clone 28D4; BD Biosciences), anti‐CD105‐BV786 (clone 266; BD Biosciences) and anti‐CD73‐BUV737 (clone AD2; BD Biosciences). After washing twice in PBS, cells were incubated with a cocktail of fixable viability stain 575V and DRAQ5 for 15 min at RT and then washed in PBS, resuspended in 1% formaldehyde (Sigma‐Aldrich) and stored at 4°C until flow cytometric analysis the next day.

Samples were acquired on an LSR Fortessa Special Order Research Product, using FACS Diva Software version 8.0 and equipped with 355 nm (20 mW), 405 nm (50 mW), 488 nm (50 mW), 561 nm (50 mW) and 633 nm (40 mW) lasers (BD Biosciences). Analysis was performed using FCS Express v4 Flow Research Edition (De Novo Software, Pasadena, CA, USA).

### Single‐cell transcriptomics

Cryopreserved dissociated tumor specimens were thawed rapidly in a 37°C water bath and washed in 9 mL cold MACS buffer (PBS+ 0.5% FBS). Contaminating myelin debris was removed using Myelin Removal Beads II (Miltenyi), according to the manufacturer’s protocol using the LS column option. As a result of the vast excess of myelin debris, two rounds of depletion were performed. Single‐cell libraries were generated with the Chromium™ Single Cell 3’ Library & Gel Bead Kit V3 (10X Genomics, Pleasanton, CA, USA) as per the Chromium™ Single Cell 3’ Library protocol User Guide (CG000183, Revision A). In order to capture ~ 10 000 cells per sample (at an approximate 65% capture efficiency), 16 000 cells were loaded for each sample. After GEM generation on the Chromium™ Controller, and subsequent GEM‐RT, the cDNA was recovered with 10X Genomics Recovery Agent and Dynabeads MyOne SILANE (PN 2000048) and subjected to PCR under the following conditions: 98°C for 3 min, 11 cycles of 98°C for 15 s, 63°C for 20 s, 72°C for 1 min, and a final step of 72°C for 1 min. The 0.6X SPRI Bead cleaned (Beckman Coulter, Brea, CA, USA) cDNA then underwent fragmentation, end‐repair and A‐tailing and a double‐sided SPRI Bead size selection. Once the adaptor ligation and final Sample Index PCR and SPRI bead clean‐up were complete, libraries were pooled in equimolar ratio for a final 4 nM sequencing pool. This library was then denatured and diluted to a final concentration of 1.5 pM (Illumina Denature and Dilute Library Guide protocol #15048776, v09), and loaded onto a 150 cycle High Output Nextseq kit for paired‐end sequencing (Read 1 = 26 cycles, i7 index = 8 cycles, i5 index = 0 cycles, Read 2 = 98 cycles). Library and cDNA quality control were carried out on the Agilent Bioanalyser 2100 Instrument using a High Sensitivity kit (Agilent Technologies, Santa Clara, CA, USA).

Sequenced single‐cell libraries were initially processed using bcl2fastq (Illumina, San Diego, CA, USA) following protocols as detailed by 10X Genomics, to generate raw read files. Alignment of raw reads to the human pre‐mRNA reference genome (hg19) and subsequent generation of gene expression matrices was carried out using the Cell Ranger count pipeline version 3.1.0 (10X Genomics). The resultant files were imported into R version 3.6.3 and processed using Seurat version 3.1.5^78^ for quality control, normalisation, integration, variable gene selection, dimensionality reduction, clustering and visualisation. In brief, cells were excluded if one or more of the following thresholds were met: (1) cells expressed fewer than 200 genes, (2) the number of expressed genes deviated outside ± 2 standard deviations from the mean and (3) the detected mitochondrial gene fraction exceeded 10%. The remaining cells were log‐normalised by total expression and scaled to 10,000 transcripts per cell using the NormalizeData function. Cells from different libraries were combined and harmonised by assessing the pairwise correspondence between a set of representative genes (anchors) between all pairs of libraries using the FindIntegrationAnchors (dims = 1:30, k.filter = 200) and IntegrateData (dims = 1:30) functions. Variable genes were identified with the FindVariableGenes function using vst as the selection method and returning 2000 features. Using only the subset of variable features, the data were then auto‐scaled and summarised by principal component analysis using the ScaleData and RunPCA functions. Subsequent graph‐based clustering of cells according to their gene expression profile was achieved using the FindNeighbors and FindClusters functions of Seurat with resolution set to 0.5. UMAP plots were generated using the first 20 principal components. Clusters were annotated manually based on known sets of cell‐specific markers. Identification of positive marker genes for each cluster was performed by pairwise differential gene expression analysis for each cluster against all other clusters using the FindAllMarkers function setting min.pct = 0.25 and logfc.threshold = 0.25.

### Additional bioinformatics analyses

To analyse The Cancer Genome Atlas (TCGA) data, RNA sequencing (RNA‐seqV2), microarray and clinical (Biotab) data for the glioblastoma multiforme (GBM) and low‐grade glioma (LGG) cohorts were downloaded from the Data Portal at http://cancergenome.nih.gov/. Data were analysed in R using Bioconductor to extract expression values for *FAP* and overall survival time for each patient, with downstream analyses conducted in GraphPad Prism v5.

Expression patterns of *FAP* in healthy normal tissues were examined by analysing RNA‐sequencing data from the Genotype‐Tissue Expression (GTEx) Project. The graph presented in Figure 1d was generated using the gene expression viewer on the GTeX Portal on 27/11/2019 and represents data from GTEx Analysis Release V8 (dbGaP Accession phs000424.v8.p2). Expression values are calculated as transcripts per million (TPM) from a gene model with isoforms collapsed to a single gene, with no other normalisation steps applied.

Expression patterns of *FAP* in different areas of glioblastoma tissues (Figure 4a) were determined by analysis of Ivy Glioblastoma Project data^38^, which used laser microdissection followed by RNA sequencing of distinct micro‐anatomical regions within 10 glioblastoma tumors. Gene‐level fragments per kilobase per million (FPKM) values, subjected to z‐score normalisation, were downloaded from the Ivy GAP data portal on 13/6/2018.

Expression of *FAP* in cultured cell lines (Figure 3a) was assessed using microarray data from the Cancer Cell Line Encylopedia (GSE36133),^33^ while *FAP* expression in paediatric brain tumors (Figure 7) was assessed using microarray dataset GSE50161.^48^ Datasets were analysed in R to extract expression values for *FAP*.

### Statistical analyses

Statistical analysis and graphing were conducted using GraphPad Prism v5.04. Pairwise comparisons were conducted using the Mann–Whitney *U*‐test, while group comparisons were conducted using the Kruskal–Wallis test with Dunn’s post‐test. Survival analyses were conducted using Kaplan–Meier analysis and the curves were compared using the log‐rank test. For all tests, *P*‐values under 0.05 were considered significant (*, *P* < 0.05; **, *P* < 0.01; ***, *P* < 0.001; ns, not significant).

## Author Contribution

**Lisa Ebert:** Conceptualization; Data curation; Formal analysis; Funding acquisition; Investigation; Methodology; Project administration; Supervision; Writing‐original draft; Writing‐review & editing. **Wenbo Yu:** Conceptualization; Investigation; Writing‐review & editing. **Tessa Gargett:** Conceptualization; Formal analysis; Investigation; Methodology; Project administration; Supervision; Writing‐review & editing. **John Toubia:** Data curation; Formal analysis; Methodology; Supervision; Visualization. **Paris M Kollis:** Data curation; Formal analysis; Methodology. **Melinda N Tea:** Data curation; Investigation; Project administration; Resources; Writing‐review & editing. **Brenton Wayne Ebert:** Data curation; Formal analysis; Visualization. **Cedric Bardy:** Conceptualization; Funding acquisition; Methodology; Resources; Writing‐review & editing. **Mark van den Hurk:** Data curation; Formal analysis; Investigation; Methodology; Resources; Visualization; Writing‐review & editing. **Claudine S Bonder:** Conceptualization; Funding acquisition; Methodology; Resources; Supervision; Writing‐review & editing. **Jim Manavis:** Data curation; Investigation; Methodology; Resources. **Kathleen S Ensbey:** Investigation; Methodology. **Mariana Oksdath Mansilla:** Conceptualization; Investigation. **Kaitlin Scheer:** Conceptualization; Investigation. **Sally L Perrin:** Conceptualization; Investigation; Writing‐review & editing. **Rebecca J Ormsby:** Data curation; Funding acquisition; Methodology; Project administration; Resources; Writing‐review & editing. **Santosh Poonnoose:** Conceptualization; Funding acquisition; Methodology; Project administration; Resources; Supervision. **Barbara Koszyca:** Investigation; Resources. **Stuart Pitson:** Funding acquisition; Project administration; Resources; Supervision; Writing‐review & editing. **Bryan W Day:** Conceptualization; Data curation; Funding acquisition; Investigation; Methodology; Resources; Supervision; Writing‐review & editing. **Guillermo A Gomez:** Conceptualization; Data curation; Formal analysis; Funding acquisition; Project administration; Resources; Supervision; Writing‐review & editing. **Michael Brown:** Conceptualization; Funding acquisition; Project administration; Resources; Supervision; Writing‐review & editing.

## Conflict of interest

The authors declare no conflict of interest.
