# Supplementary material for: Endothelial, pericyte and tumor cell expression in glioblastoma identifies fibroblast activation protein (FAP) as an excellent target for immunotherapy
Source: Clin Transl Immunology. 2020 Oct 14;9(10):e1191. doi: 10.1002/cti2.1191 (PMC7557106; doi:10.1002/cti2.1191)
Supplement: Supplementary file 1 [file CTI2-9-e1191-s001.pdf]

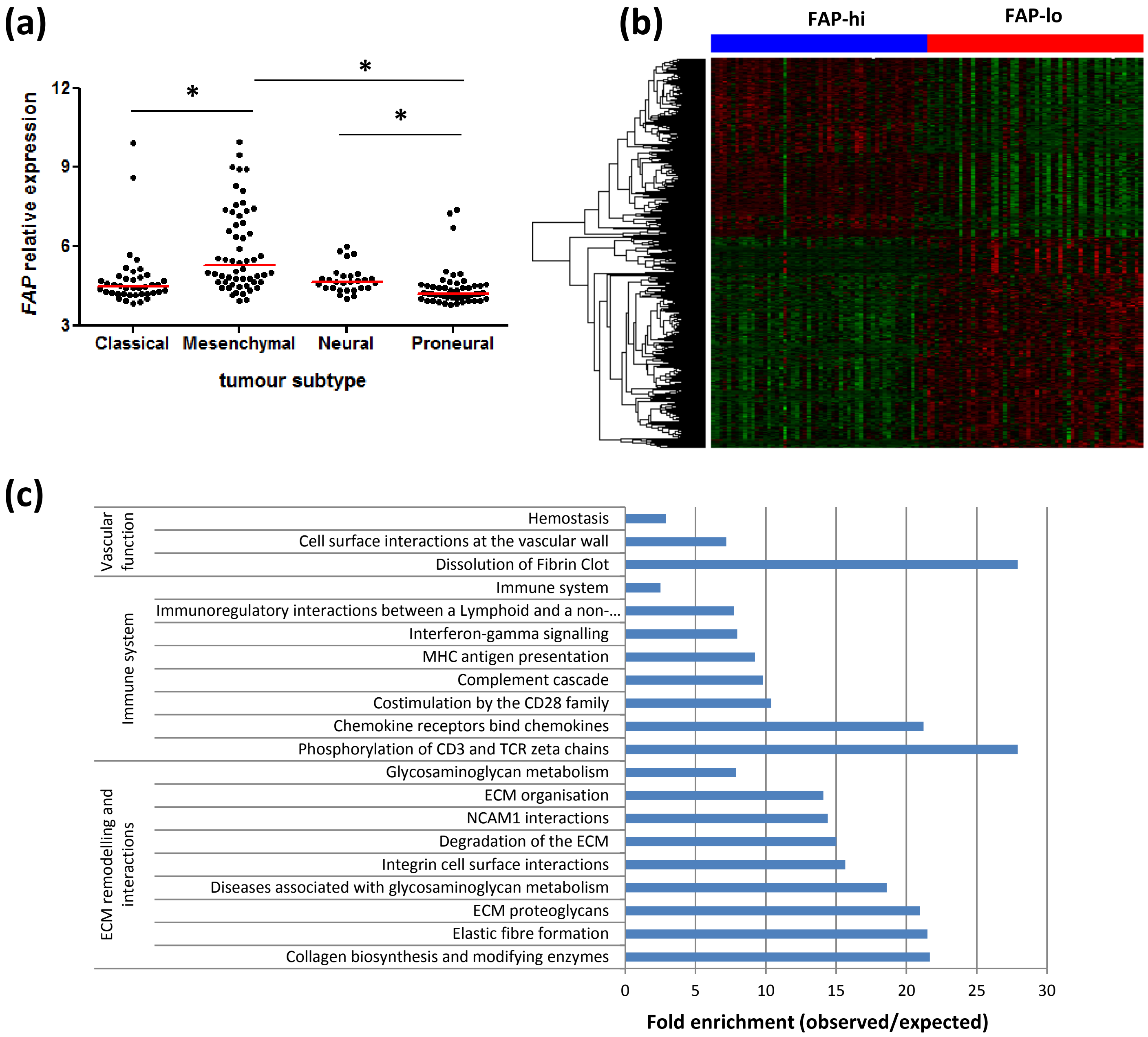

<u>Supplementary figure 1</u> – *FAP* overexpression associates with the mesenchymal phenotype and genes associated with invasion, inflammation and vascular function. Microarray data from TCGA GBM dataset was analysed using R. **(a)**: Glioblastoma specimens were divided into 4 subtypes on the basis of multi-gene signatures and *FAP* expression compared. Differences between the groups were significant by Kruskal-Wallis test ($p < 0.0001$); pair-wise comparisons significant by Dunn's post-test are indicated by asterisks. **(b)**: Heatmap showing genes differentially expressed between *FAP*-hi and *FAP*-lo tumours; a total of 865 genes showed >2-fold difference between the groups, using an FDR cut-off of <0.05. **(c)**: To identify gene pathways associated with high *FAP* expression in tumours, the list of genes most overexpressed (>3-fold) by the *FAP*-hi group was analysed by PANTHER Overrepresentation test using the Reactome Pathways annotation and Fisher's Exact test with FDR multiple test correction. All results have FDR < 0.05. Pathways associated with high *FAP* expression are categorised into 3 functional subsets, and the fold enrichment of each pathway (observed/expected) is shown.

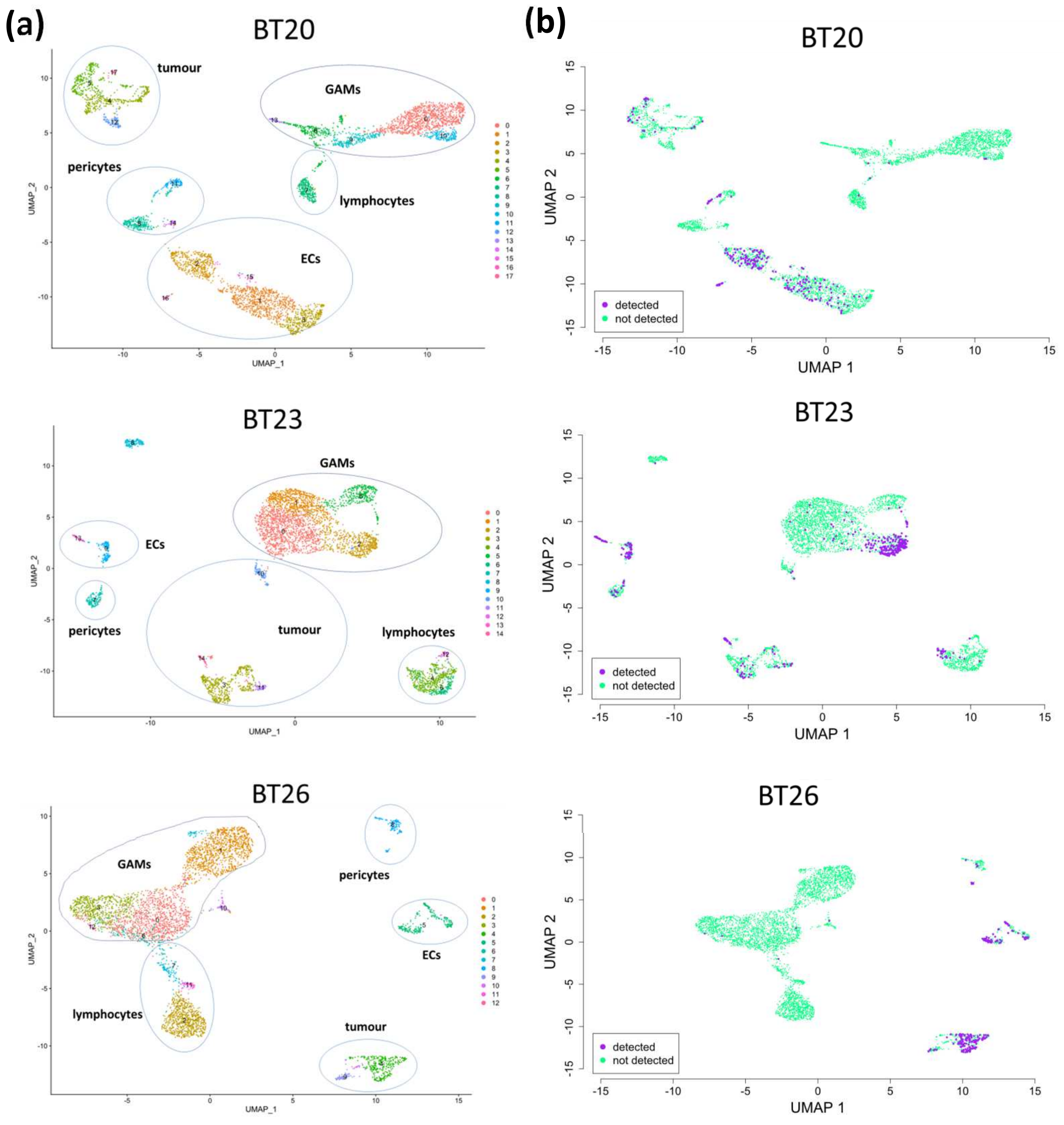

<u>Supplementary figure 2</u> – ***Single cell transcriptomic analysis of three glioblastoma specimens***. Fresh tumour tissue specimens from three patients (BT20, BT23, BT26) were dissociated to single cell suspensions and analysed by scRNAseq. **(a):** UMAP plots for each specimen showing unsupervised clustering of cells, with the cell type of each cluster annotated according to the presence of marker genes. **(b)**: UMAP plots were coloured according to whether *FAP* transcripts were expressed (purple) or not (green) in individual cells.
