# Supplementary material for: Endothelial, pericyte and tumor cell expression in glioblastoma identifies fibroblast activation protein (FAP) as an excellent target for immunotherapy
Source: Clin Transl Immunology. 2020 Oct 14;9(10):e1191. doi: 10.1002/cti2.1191 (PMC7557106; doi:10.1002/cti2.1191)
Supplement: Supplementary file 2 [file CTI2-9-e1191-s002.pdf]

Supplementary table 1: Characteristics of FFPE glioblastoma specimens and outcome of FAP IHC

Patient	Gender	Primary/ recurrence	Diagnosis/pathology details	FAP IHC result		
				Breadth	Intensity	IRS ‡
1	M	Primary	Glioblastoma	1.5	2	3
		Recurrence	Glioblastoma	2	2	4
2	F	Primary	Glioblastoma	1	2	2
		Recurrence	Glioblastoma showing treatment-related changes	2	1	2
3	F	Primary	Glioblastoma with areas of oligodendroglial differentiation	2	3	6
3		Recurrence	Glioblastoma showing treatment-related changes and oligodendroglial differentiation	3	1	3
4	M	Recurrence	Glioblastoma showing sarcomatoid & small cell components and treatment-related changes	2	2	4
5	M	Primary	Glioblastoma	2	2	4
6	M	Primary	Glioblastoma	4	1	4
7	M	Recurrence	Glioblastoma showing treatment-related changes	1.5	1.5	2.5
8	F	Primary	Glioblastoma with prominent gemistocytic component	1	2	2
9	F	Recurrence	Glioblastoma with prominent gemistocytic component	2	3	6
10	F	Recurrence (PM) †	Glioblastoma	1	3	3
11	M	Recurrence (PM)	Glioblastoma	1	3	3
12	M	Primary	Glioblastoma	0	0	0
13	M	Primary	**Gliosarcoma**	3	2	6
14	M	Primary	**Gliosarcoma**	3	2	6
15	M	Primary	**Gliosarcoma**	3	3	9
16	F	Primary	Glioblastoma with sarcomatoid and small cell components	0	0	0
		Recurrence	Glioblastoma with sarcomatoid and small cell components	2	3	6
17	M	Primary	Glioblastoma	2	3	6
		Recurrence	Glioblastoma showing treatment-related changes	2	2.5	5
18	M	Primary	Glioblastoma	0	0	0
		Recurrence	Glioblastoma	0	0	0
19	M	Primary	Glioblastoma with areas of oligodendroglial differentiation	1	3	3
		Recurrence	Glioblastoma showing treatment-related changes	1	2.5	2.5
20	F	Primary	Glioblastoma	0	0	0
		Recurrence	Glioblastoma with prominent gemistocytic component and treatment-related changes	0	0	0
21	F	Primary	Glioblastoma with gemistocytic, sarcomatoid and small cell components	1	2	2
22	F	Recurrence	**Gliosarcoma**	3	3	9

† PM: post-mortem; ‡ IRS: ImmunoReactivity Score (obtained by multiplying score for breadth by score for intensity; see Materials and Methods for details)

Supplementary table 2: Healthy brain tissue specimen details and FAP IHC outcome

Donor	Regions examined	FAP staining in brain parenchyma	FAP staining on CD31+ vessels
SA0096	left frontal lobe, left anterior parietal lobe, left basal ganglia	Negative	Negative
SA0112	left superior frontal gyrus, left cingulate gyrus, left parietal cortex	Negative	Negative
SA0214	left middle frontal gyrus, middle temporal gyrus, left superior parietal lobule	Negative	Negative
CA4	right frontal lobe, right basal ganglia, right parietal lobe	Negative	Negative
CA5	right frontal lobe, right basal ganglia, right parietal lobe	Negative	Negative
CA6	right frontal lobe, right basal ganglia, right parietal lobe	Negative	Negative
